# Conjugated NOD1-Centered Dual Agonists Exhibit Modest In Vitro Immunomodulation but Potent In Vivo Adjuvanticity

**DOI:** 10.1021/acsbiomedchemau.6c00080

**Published:** 2026-06-15

**Authors:** Emiliano Paradiso, Špela Janež, Veronika Weiss, Ruža Frkanec, Žiga Jakopin

**Affiliations:** † Faculty of Pharmacy, 63721 University of Ljubljana, SI-1000 Ljubljana, Slovenia; ‡ Centre for Research and Knowledge Transfer in Biotechnology, University of Zagreb, 10000 Zagreb, Croatia

**Keywords:** NOD1, pattern recognition receptor agonists, conjugates, immunomodulator, adjuvant

## Abstract

Immunomodulators that simultaneously engage multiple pattern recognition receptors (PRRs) represent a promising strategy for shaping immune responses. Here we report the design and synthesis of covalently linked dual PRR agonists composed of a NOD1-selective ligand conjugated to agonists of TLR4, TLR7, or RIG-I. The resulting chimeric molecules were evaluated for receptor-specific agonist activation, *in vitro* immunomodulatory activity in human peripheral blood mononuclear cells, and *in vivo* adjuvant properties. In most cases, conjugation attenuated receptor agonist activity and reduced cytokine responses *in vitro* compared with mixtures of the corresponding unconjugated agonists. Despite modest *in vitro* activity, the conjugates, particularly the dual NOD1/TLR7 agonist, displayed robust adjuvant effects in a murine vaccination model. These results highlight the distinct immune signatures of conjugated NOD1-based dual agonists and support their development as next-generation vaccine adjuvants.

## Graphical abstract

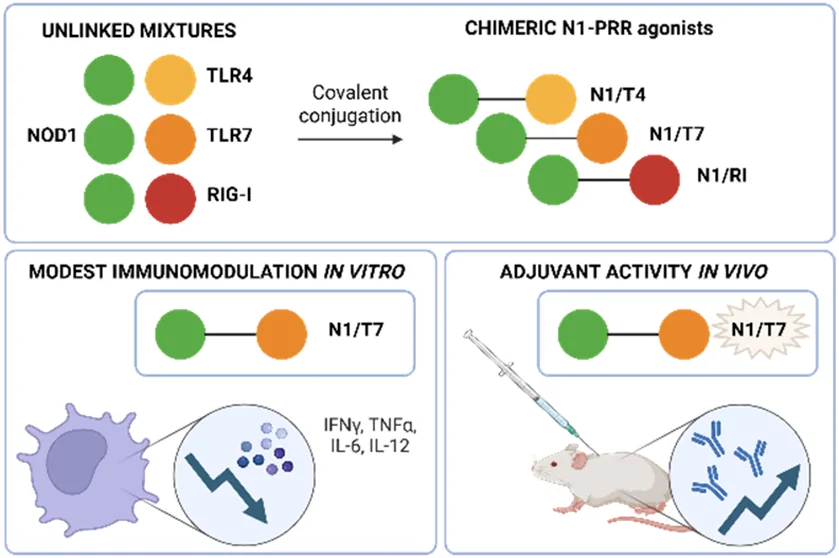

## Introduction

The innate immune system employs pattern recognition receptors (PRRs) to detect conserved pathogen-associated molecular patterns (PAMPs), thereby initiating intracellular signaling cascades that culminate in the production of inflammatory cytokines, type I interferons (IFNs), and costimulatory molecules. This process establishes a critical interface between innate and adaptive immune responses. Toll-like receptors (TLRs) predominantly monitor extracellular and endosomal compartments, whereas cytosolic sensors, including nucleotide-binding oligomerization domain-like receptors (NLRs) and retinoic acid-inducible gene I (RIG-I)-like receptors (RLRs), detect intracellular pathogenic components.^1^ Although activation of individual PRRs typically elicits moderate immune responses, extensive cross-talk among PRRs enables synergistic signal amplification and the emergence of qualitatively distinct immune signatures.^2^

Structure–activity relationship (SAR) studies have established that exposure of the diaminopimelic acid (DAP) scaffold is critical for NOD1 activation, whereas modifications at the terminal carboxyl group are generally well tolerated, allowing rational structural diversification of NOD1 ligands. Notably, *N*-lauroylation of the d-Glu α-amino group in iE-DAP analogs markedly enhances NOD1 agonistic potency, by several hundred-fold, primarily by improving membrane permeability and receptor engagement efficiency.^3^ A number of biologically significant synergistic interactions between NOD1 and other PRRs located in different subcellular compartments have been reported.^4–6^ Notably, coactivation of NOD1 with TLRs or RLRs results in enhanced pro-inflammatory and antiviral responses mediated through convergent activation of nuclear factor κB (NF-κB), JNK, and CARD-dependent pathways,^7,8^ some of which have translated successfully into *in vivo* models.^9^ For example, NOD1 has been shown to enhance antiviral immunity by directly binding viral RNA and facilitating the interaction between the RIG-I-like receptor MDA5 and its adaptor MAVS, thereby promoting antiviral gene expression and suppressing viral replication.^4,10^ Moreover, the synergistic interplay between NOD1 and TLR4 has been more extensively characterized and also constituted a central focus of our previous investigations.^11,12^ Co-stimulation of NOD1 and TLR4 in human peripheral blood mononuclear cells (PBMCs) resulted in synergistic upregulation of pro-inflammatory cytokines, including IFN-γ, tumor necrosis factor-α (TNF-α), and interleukin-6 (IL-6), and significantly enhanced natural killer (NK)-cell cytotoxic function. In parallel, cross-talk between NOD1 and TLR7 has also been observed, leading to amplified innate immune signaling through coordinated activation of NF-κB, mitogen-activated protein kinases (MAPK), and interferon regulatory factor 7 (IRF7) pathways, ultimately augmenting cytokine and interferon production.^8,13^

Chemical conjugation of distinct PRR agonists has emerged as a promising strategy to further enhance immunostimulatory activity by enabling the codelivery of multiple immunostimulatory signals to the same immune cell, potentially promoting coordinated receptor engagement and downstream signaling.^14^ As discussed above, multiple independent studies have demonstrated that simultaneous stimulation of NOD1 together with TLR4-, TLR7-, or RIG-I-associated signaling pathways can enhance innate immune activation and modulate downstream adaptive immune responses, thus providing a mechanistic rationale for the development of conjugates composed of NOD1 ligands and corresponding PRR agonists. Importantly, these studies primarily relied on coadministration of unconjugated agonist mixtures, leaving unresolved whether covalent linkage of individual PRR agonists may alter the spatial and temporal coordination of receptor activation and consequently modulate the resulting immune response. Based on these observations and our prior work on NOD2 agonist-based conjugates,^15,16^ we sought to investigate whether conjugation of NOD1 agonists to structurally and functionally distinct PRR ligands could generate immunological profiles differing from those induced by unconjugated agonist combinations. We report here the construction of a novel class of conjugated dual NOD1/PRR agonists prepared through a straightforward amide coupling strategy. To the best of our knowledge, conjugates featuring a NOD1 ligand as a central scaffold have not been reported to date. Herein we investigate how the identity of the PRR agonist (TLR4, TLR7, and RIG-I) tethered to the central NOD1 ligand influences receptor-specific agonist activity, *in vitro* immunomodulatory profiles, and *in vivo* adjuvant properties.

## Results and Discussion

### Design and Synthesis

To enable the attachment of carboxylic acid-decorated PRR agonists, a modified analog of C12-iE-DAP (Figure 1A) bearing an amino-handle group at the C12 position has been synthesized using a slightly modified literature procedure.^3^ This structural modification preserves the pharmacophoric features of the parent compound while providing a convenient aliphatic spacer for conjugate assembly. The agonists of RIG-I,^17^ TLR4,^18^ and TLR7 ^19^ (Figure 1B) employed as the second building blocks were synthesized according to reported procedures. These compounds were selected based on their synthetic accessibility and the presence of free carboxylic acid functionalities enabling covalent conjugation. The central design principle of these constructs relies on the presumed *in vivo* cleavage of the amide bond, which may allow the release of the NOD1 and PRR agonists to act independently on their respective receptors. At the same time, intact conjugates may retain partial agonistic activity prior to linker hydrolysis, as previously observed for related systems.^20–22^

**Figure 1:**
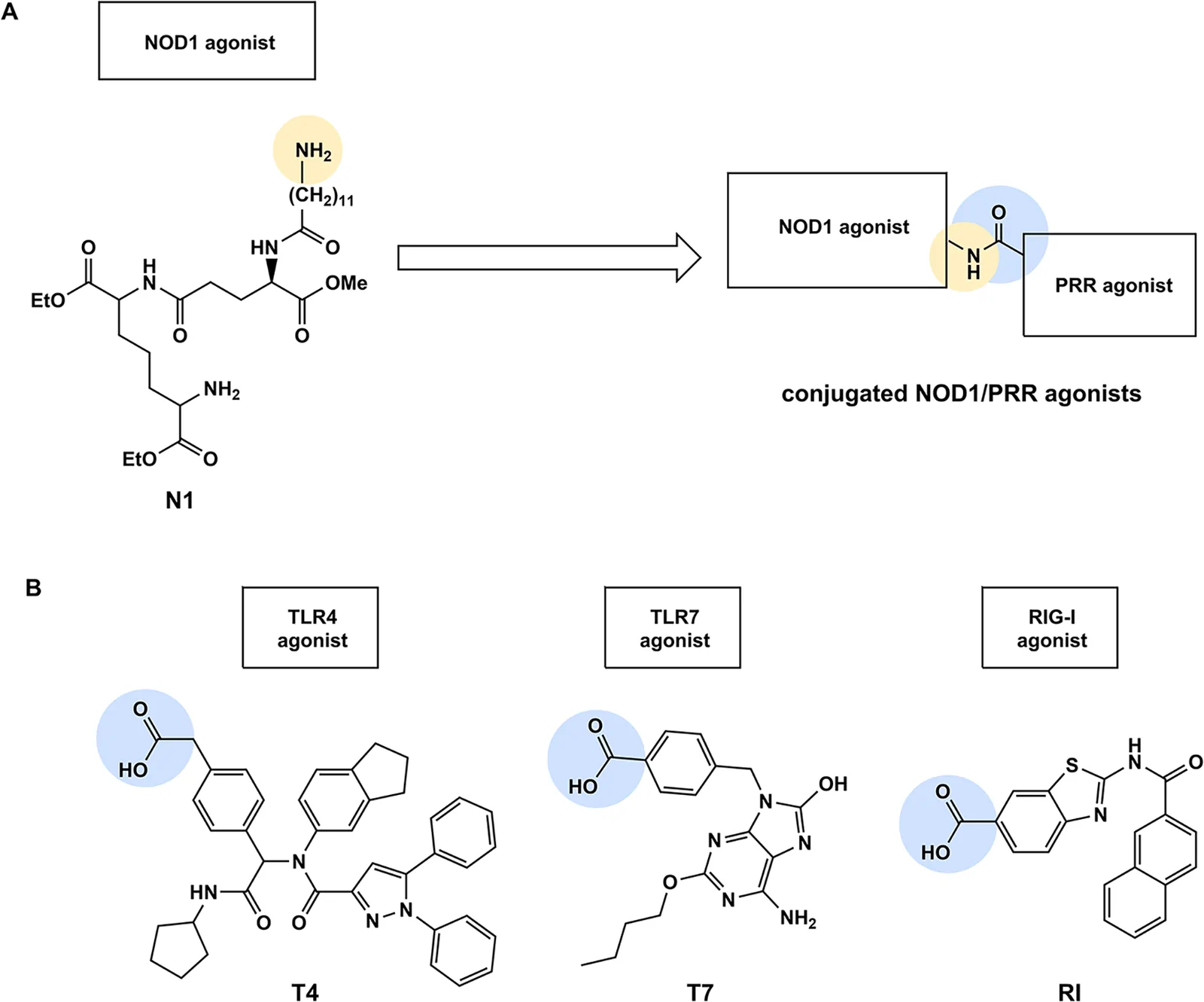
(A) Design of chimeric NOD1 ligand-featuring conjugates; (B) selected PRR agonists employed as building blocks for this work.

As depicted in Scheme 1, we synthesized a focused library of chimeric conjugates composed of the NOD1 ligand **N1**, an amino group-decorated derivative of C12-iE-DAP, and small molecule agonists of TLR4 (**T4**), TLR7 (**T7**), and RIG-I (**RI**). The monoprotected Boc derivative of DAP **1** was prepared as described in the literature and coupled to orthogonally protected *Z*-d-GluOMe.^3^ Subsequent catalytic hydrogenation removed the Cbz protecting group to afford the free amine **2**. This intermediate was coupled with Cbz-protected 12-aminolauric acid **3**, followed by catalytic hydrogenation to yield the desired NOD1 ligand building block bearing a free amino handle (**4**). Compound **5** was obtained through direct amide coupling of the Boc-protected NOD1 ligand **4** with the carboxylic acid group of the TLR4 agonist (**T4**), using HATU in the presence of *N*,*N*-diisopropylethylamine (DIPEA) and catalytic 4-dimethylaminopyridine (DMAP). The same procedure was successfully applied to generate the corresponding conjugate precursors with TLR7 (**T7**) and RIG-I (**RI**) agonists, affording intermediates **6** and **7**. Finally, acidolytic removal of the Boc protecting group (TFA/DCM) yielded the target conjugates **N1/T4** (NOD1/TLR4 ligand), **N1/RI** (NOD1/RIG-I ligand), and **N1/T7** (NOD1/TLR7 ligand), each bearing a free ε-amine group, required for NOD1 recognition and biological activity. For comparison in subsequent biological assays, compound **4** was also subjected to Boc deprotection to furnish the individual NOD1 ligand **N1** (i.e., 12-amino derivative of C12-iE-DAP).

**Scheme 1:**
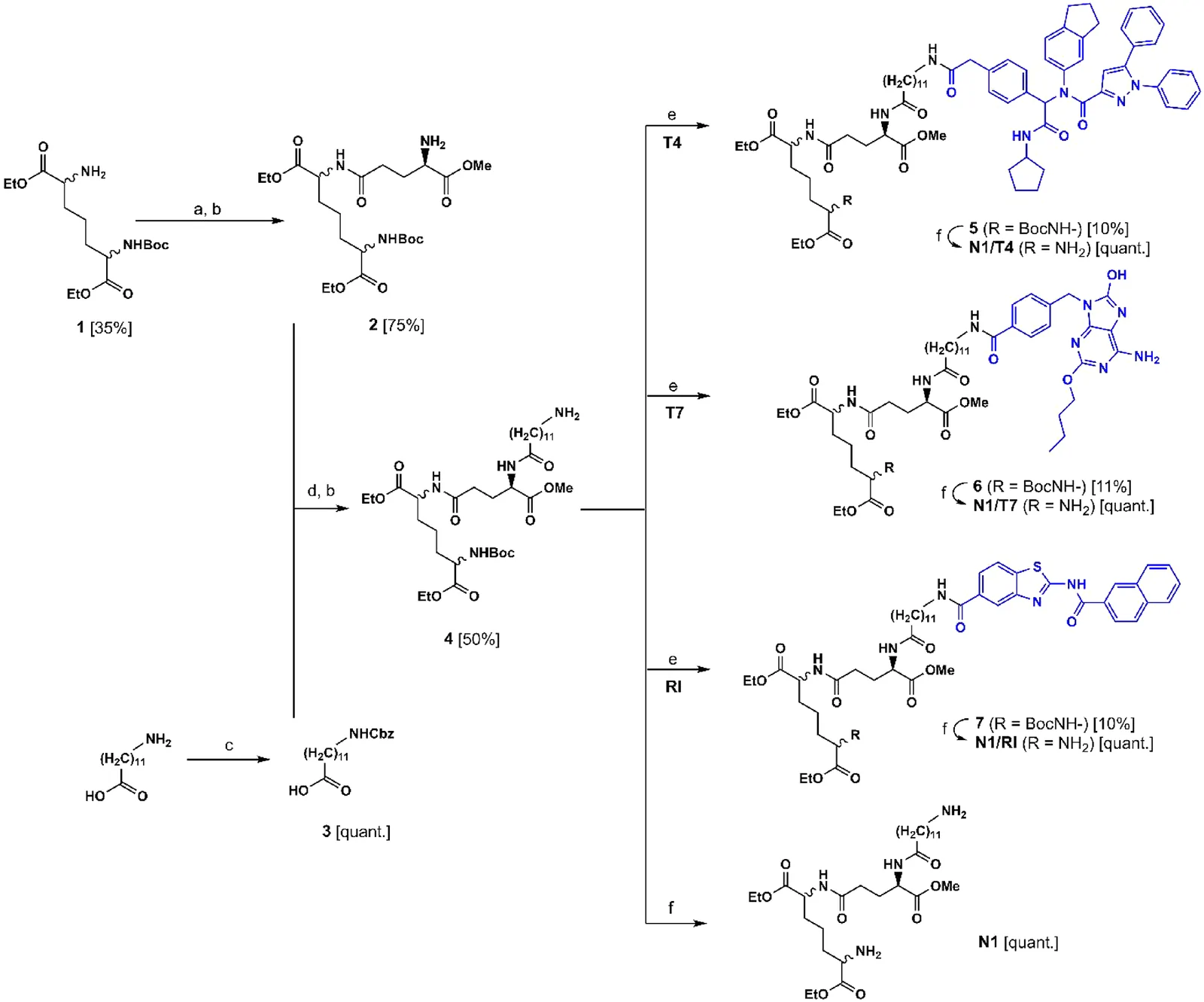
Synthesis of Conjugated NOD1/PRR Agonists and the Amino-Derivative of C12-iE-DAP, NOD1 Agonist **N1** ^a^

a Reagents and conditions: (a) (*4S*)-4-{[(benzyloxy)­carbonyl]­amino}-5-methoxy-5-oxopentanoic acid, EDC/HOBt, DIPEA, DMAP, DMF, 0 °C to rt, 16 h; (b) H_2_, Pd/C 10 wt %, 6h; (c) CbzCl, 1 M NaHCO_3_ (aq), dioxane, 0 °C; (d) EDC/HOBt, DIPEA, DMAP, DMF, 0 °C to rt, 16 h; (e) HATU, DIPEA, DMF, DMAP, 0 °C to rt, 16 h; (f) TFA/DCM (1:5), rt, 4 h.

### Biological Evaluation

The synthesized conjugates were evaluated for receptor-specific activity using commercially available HEK-Blue and HEK-Lucia reporter cell lines. HEK-Blue cells stably express an NF-κB-inducible secreted embryonic alkaline phosphatase (SEAP) reporter gene, whereas HEK-Lucia cells express a luciferase reporter gene, enabling real-time monitoring of receptor activation. Cytotoxicity profiling using an MTS assay confirmed the absence of overt toxicity for all tested conjugates at the highest concentrations used in the reporter assays (Figure S1).

Quantitative assessment of NOD1 activation was performed by stimulating HEK-Blue hNOD1 cells with the synthesized conjugates across a broad concentration range (0.005–20 μM) (Figure 2). Among the conjugates, **N1/T4** displayed the strongest apparent NOD1 activation profile in the reporter assay and retained substantial activity following conjugation, comparable to that of the individual NOD1 ligand **N1**. Thus, conjugation to the TLR4 agonist only marginally affected NOD1 activity. In contrast, a more pronounced reduction in potency was observed for **N1/RI** and **N1/T7**, which exhibited significant NOD1 activation only at the two highest concentrations tested (1.25 and 20 μM). Importantly, hydrolysis of the amide bond linking the two building blocks under the assay conditions is unlikely, as previously demonstrated in HEK-Blue cell models.^15,23^

**Figure 2:**
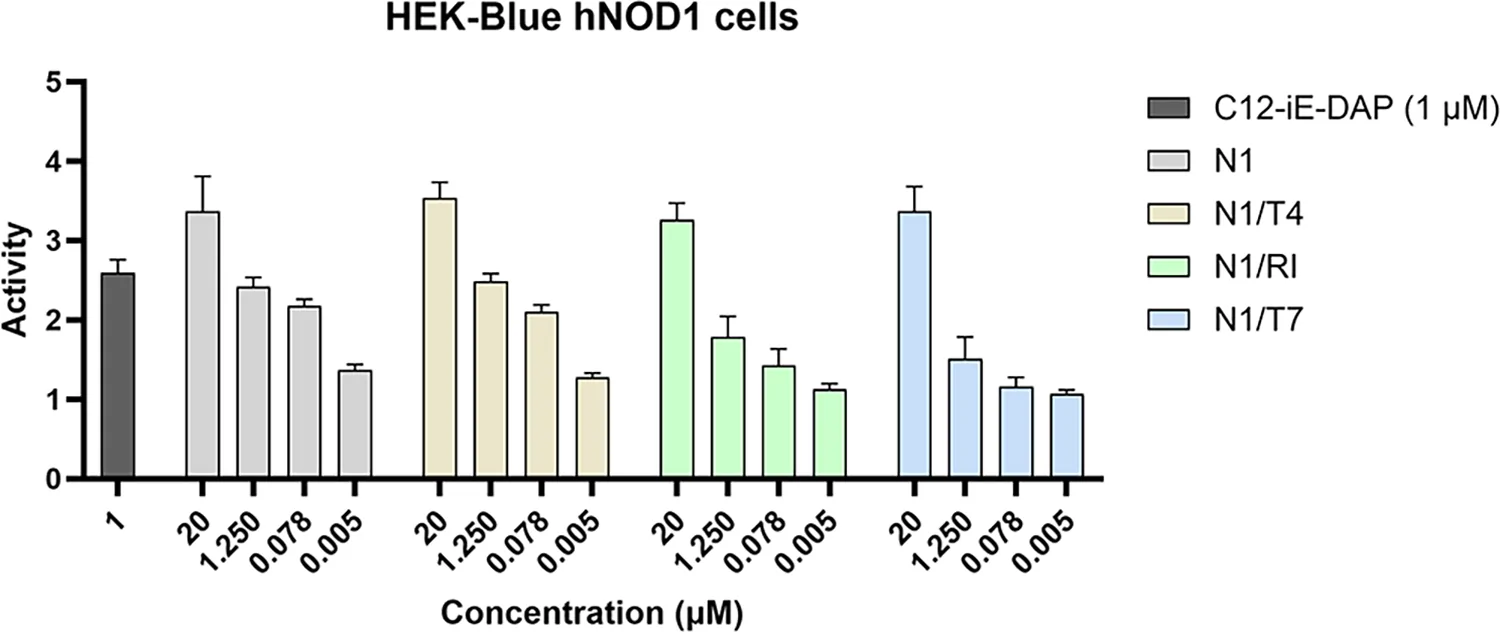
Concentration-dependent NOD1 agonist activities of conjugated NOD1/PRR agonists **N1/T4**, **N1/RI**, and **N1/T7** compared to individual NOD1 ligand **N1**. SEAP activities were measured in HEK-Blue hNOD1 cell supernatants after incubation for 18 h with compounds of interest at different concentrations (5 nM–20 μM). The data are shown as fold increases of NF-κB transcriptional activity relative to the negative control (0.1% DMSO) and are expressed as means ± SEM of at least three independent experiments.

Dose–response experiments were also conducted in additional reporter cell lines using concentration ranges appropriate for the intrinsic potency of the corresponding PRR agonists (Figure 3). TLR4 activity for **N1/T4** was evaluated at 0.5–5 μM, TLR7 activity for **N1/T7** at 10 nM–2.5 μM, and RIG-I activity for **N1/RI** at 1–100 μM. Overall, conjugation produced nuanced effects on receptor activation, predominantly affecting potency rather than maximal efficacy. For example, **N1/T4** retained TLR4 agonism at all tested concentrations and achieved a maximal response comparable to that of the parent agonist **T4**, although with reduced apparent potency. Similarly, conjugation through the benzylic carboxylate of the TLR7 agonist reduced the potency of **N1/T7** relative to the individual agonist **T7**, while maintaining similar maximal activity. In contrast, the weak RIG-I activity of the parent agonist **RI**, observed only at the highest concentration tested, was modestly enhanced upon conjugation to the NOD1 ligand. In general, the 12-amino group in **N1** proved to be a viable attachment site for decoration with other PRR agonistic moieties since conjugation did not abolish the individual receptor agonist activities and all conjugates retained measurable receptor-activating properties within the concentration ranges subsequently employed in the functional assays.

**Figure 3:**
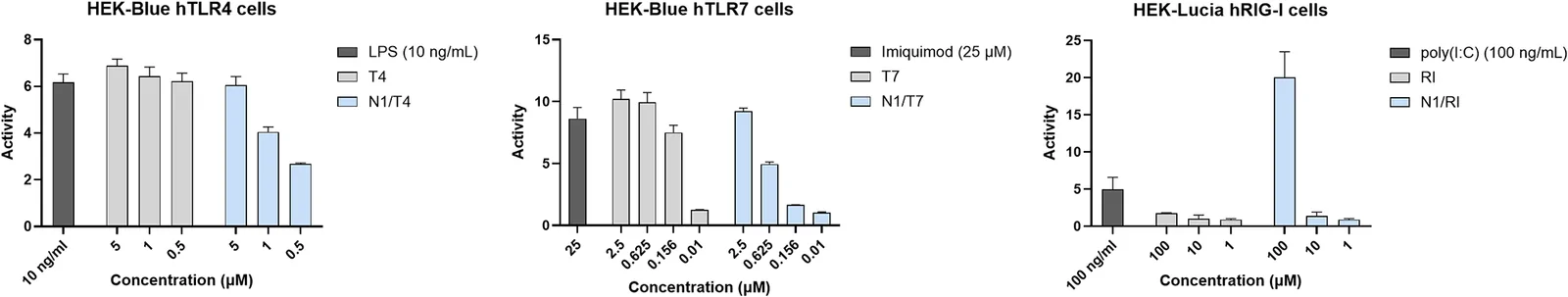
Concentration-dependent receptor agonist activities of conjugated NOD1/PRR agonists compared to bona fide commercially available ligands of corresponding PRRs. SEAP activities were measured in HEK-Blue hTLR4, HEK-Blue hTLR7 and HEK-Lucia RIG-I cell supernatants after incubation for 18 h with compounds of interest at different concentration ranges. The data are shown as fold increases of NF-κB transcriptional/luciferase activity relative to the negative control (0.1% DMSO) and are expressed as means ± SEM of at least three independent experiments.

To characterize the immune fingerprints of the conjugates, we next evaluated their immunomodulatory activity in PBMCs, a heterogeneous population of primary immune cells. Cells were treated for 18 h with compounds or compound mixtures at concentrations of 1 and 10 μM. Cytokine secretion was quantified using a multiplex immunoassay, enabling a direct comparison between the individual PRR ligands, conjugated PRR ligands and their unconjugated counterparts (Figures 4 (10 μM) and S2 (1 μM)); the obtained data revealed several notable trends. First, the activity of the unconjugated **N1** + **T4** mixture was not substantially greater than the apparent combined contributions of the individual agonists, particularly the intrinsically potent TLR4 agonist **T4** itself, across all cytokines. Under the tested conditions, a pronounced synergistic effect was therefore not evident for this agonist pair. In contrast, the biological responses induced by the **N1** + **T7** mixture exceeded the apparent additive contributions of the individual agonists in case of IL-1β, IL-6, TNF-α, and IFNγ. Moreover, the **N1** + **RI** mixture nor any of the individual agonists induced a significant cytokine release, thus indicating lack of cross-activation. As for the conjugates, consistent with the reporter assay results, the NOD1/TLR4 conjugate **N1/T4** induced the most pronounced cytokine response at 10 μM, stimulating the secretion of pro-inflammatory cytokines and chemokines including IL-1β, IL-6, IL-10, and monocyte chemoattractant protein-1 (MCP-1). However, these responses remained lower than those elicited by the unlinked mixture of NOD1 and TLR4 agonists (**N1** + **T4**). At 1 μM, the pro-inflammatory profile of **N1/T4** was markedly attenuated, with only a modest increase in MCP-1 secretion, indicating a clear dose-dependent effect. The reduced cytokine induction likely reflects partial impairment of receptor engagement resulting from steric constraints or altered cellular uptake associated with conjugation.

**Figure 4:**
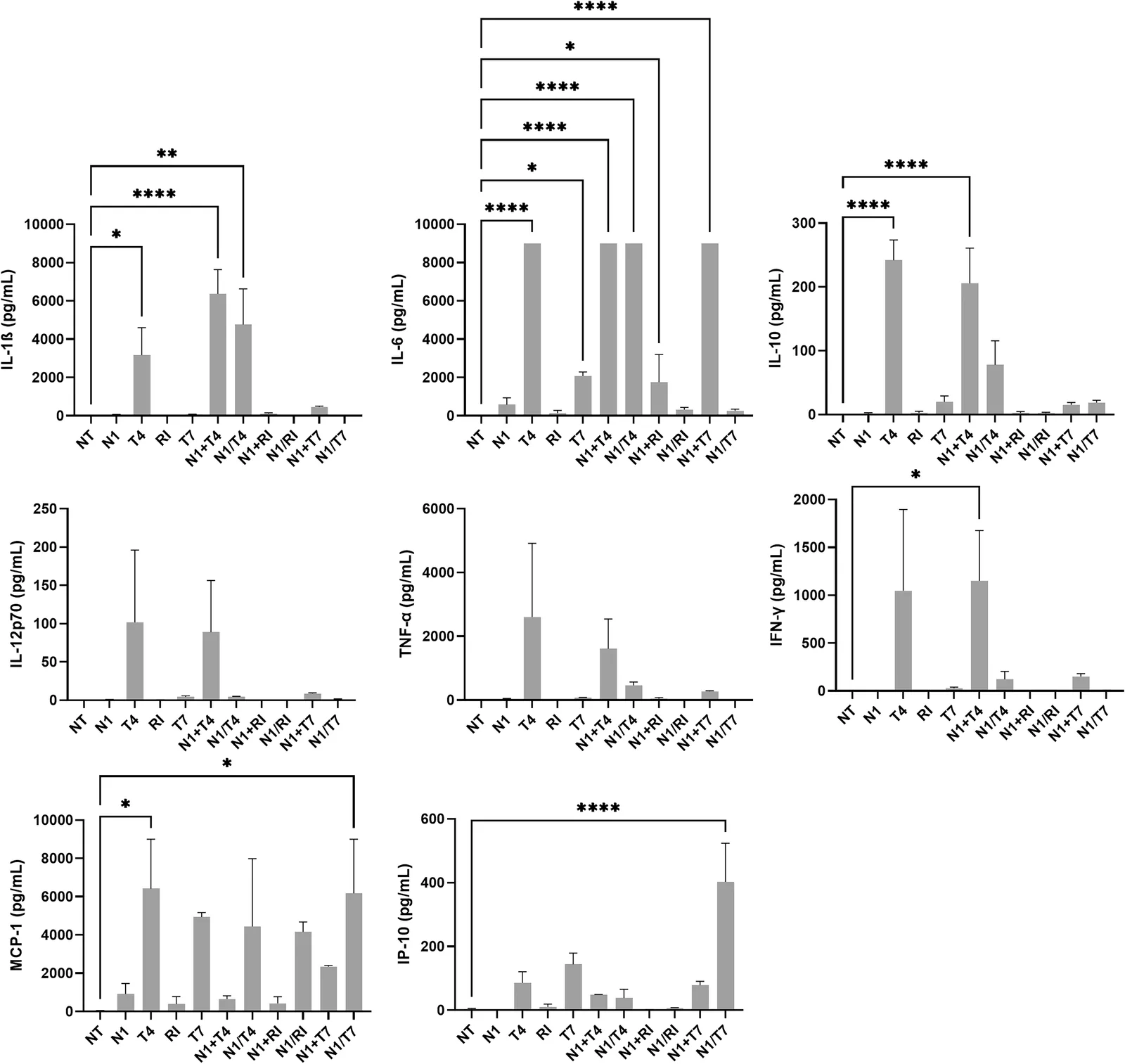
Cytokine profiling of PBMCs stimulated with tested compounds at 10 μM. Cytokine concentrations were measured after 18 h stimulation with unlinked mixtures of corresponding agonists, conjugated agonists or control vehicle (0.1% DMSO). Data are expressed as mean ± SEM of 2 or 3 independent experiments. *, *p* ≤ 0.05, **, *p* ≤ 0.01, ***, *p* ≤ 0.001, ****, *p* ≤ 0.0001 versus control (one-way ANOVA post hoc Dunnett’s tests).

In contrast, although the NOD1/TLR7 conjugate **N1/T7** produced only limited induction of classical pro-inflammatory cytokines, it triggered a pronounced increase in IFN-γ-induced protein (IP-10; CXCL10) and MCP-1 secretion at 10 μM, exceeding the levels observed for the corresponding mixture of unconjugated agonists. This finding is noteworthy because IP-10 plays a central role in cellular immunity, acting as a chemoattractant for activated T cells, regulating immune cell trafficking, and modulating inflammatory responses. Emerging evidence suggests that IP-10 may be particularly valuable in the context of immunotherapy.^24^ The NOD1/RIG-I conjugate **N1/RI** exhibited the weakest immunomodulatory profile, producing only a modest increase in MCP-1 secretion. Notably, the unlinked mixture of NOD1 and RIG-I agonists also failed to induce a significant cytokine response at both tested concentrations. Overall, chemical conjugation tended to attenuate the activity of the individual agonists, whereas mixtures of unconjugated ligands frequently displayed enhanced immune activation, consistent with our previous findings for the combination of NOD1 and TLR4 agonists.^11^ Indeed, synergistic enhancement of TNF-α, IL-1β, IL-6, IL-10, IL-4, and GM-CSF following costimulation of TLRs and NOD1, even at subactive concentrations of TLR agonists, has been widely reported.^8,9^ The combination of NOD1 and TLR7 agonists also elicited a synergistic response in PBMCs, although less pronounced than that reported for the R848 (TLR7/8 agonist) and iE-DAP mixture.^13^ This discrepancy likely reflects the dual TLR7/8 activity of R848 and the high expression of TLR8 in monocytes, an abundant PBMC subset.

To further delineate the immunomodulatory potential of synthesized conjugates, we assessed PBMC-mediated cytotoxicity against K562 cancer cells following stimulation with the conjugates or the corresponding mixtures of unconjugated agonists; IL-2 served as a positive control (Figure 5). The PBMC/K562 assay was primarily intended as a comparative functional assessment of immune activation signatures induced by unconjugated agonist mixtures and their corresponding conjugates rather than as a rigorous quantitative synergy analysis. Overall, the conjugated NOD1/PRR agonists failed to induce substantial cytolytic activity at either 1 or 10 μM. A modest increase in cytotoxicity was observed for the NOD1/TLR7 conjugate **N1/T7** at 10 μM relative to the vehicle-treated control, although the response remained significantly lower than that elicited by the corresponding unconjugated mixture (**N1** + **T7**). Similarly, the combination of unconjugated NOD1 agonist **N1** with the TLR4 agonist **T4** (1 μM) produced cytolytic activity comparable to that induced by IL-2, a potent stimulator of NK-cell-mediated killing. Interestingly, cytolytic responses induced by both **N1/T4** and the **N1** + **T4** mixture were less pronounced at 10 μM than at 1 μM, potentially reflecting dose-dependent desensitization or off-target cellular effects that attenuate optimal immune activation. The enhancement of NK-cell cytotoxicity following NOD1 and TLR4 costimulation mirrors the trends observed in reporter assays and PBMC cytokine profiling and is consistent with our previous work.^11^

**Figure 5:**
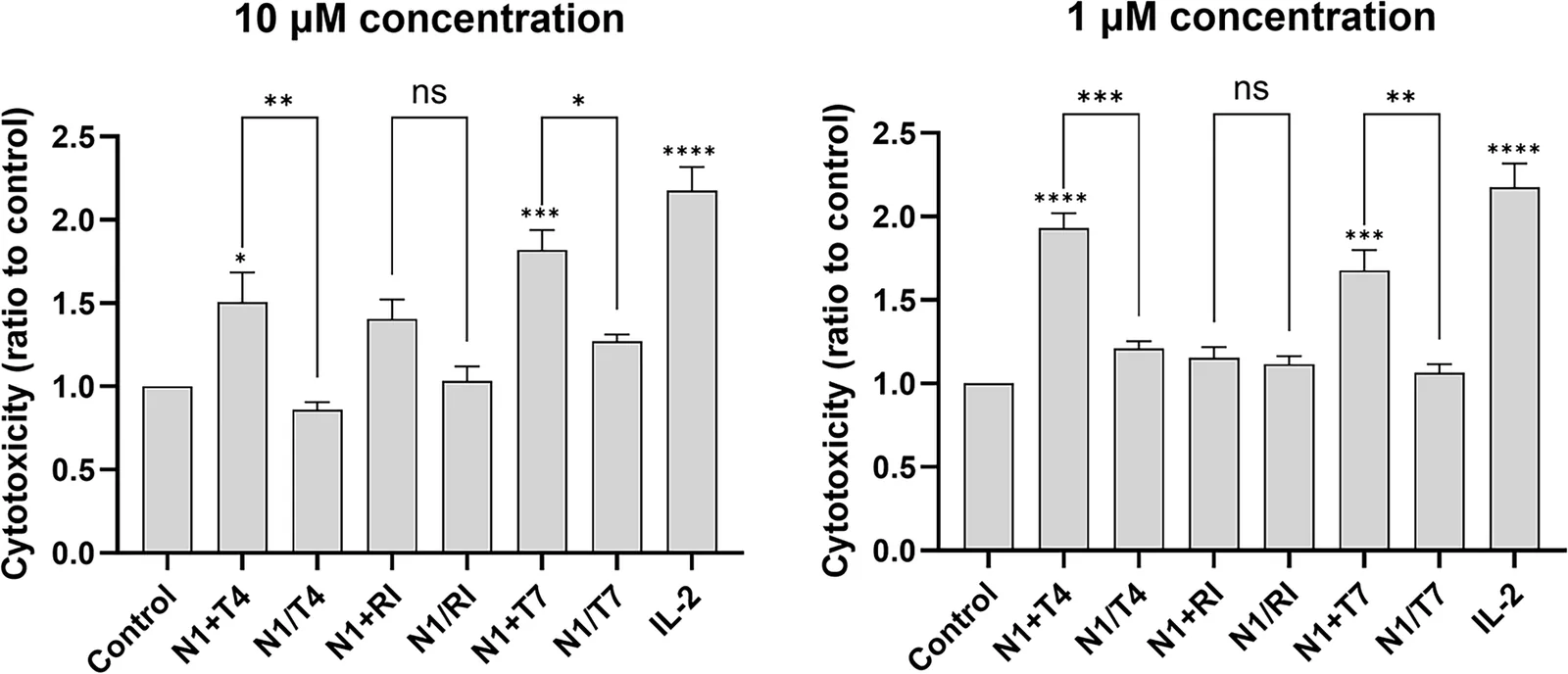
PBMC cytotoxicity against K562 cells after 18 h treatment with conjugates, unlinked mixtures (all at 1 and 10 μM), and control (IL-2, 200 U/mL), or vehicle (0.1% DMSO). Cytotoxicity was assessed after 4 h coincubation. Data are relative to the negative control (NT, 0.1% DMSO) and shown as mean ± SEM of three independent experiments. ANOVA with Bonferroni’s test compared all conditions versus control as well as unlinked mixtures to conjugates. *, *p* < 0.05, **, *p* < 0.01; ***, *p* < 0.001; ****, *p* < 0.0001.

The limited ability of the conjugated compounds to induce both cytokine release and cytolytic responses may reflect altered receptor accessibility, attenuated receptor engagement following conjugation, differences in intracellular delivery, or a combination of these factors. Direct membrane permeability measurements were not performed for the conjugates evaluated in the present study. Nevertheless, previous studies employing structurally related conjugates containing the same TLR7 and RIG-I agonists linked through analogous amide bond architectures demonstrated good stability and measurable intracellular accumulation of the conjugates in PBMC-based systems as determined by LC/MS analysis, supporting the ability of these scaffolds to access intracellular targets under biologically relevant conditions.^23^ In contrast, the corresponding unconjugated TLR7 and RIG-I agonists exhibited only minimal intracellular accumulation, consistent with the presence of free carboxylic acid functionalities expected to impair passive membrane diffusion. Notably, the contribution of membrane permeability to the observed biological activity likely differs depending on the receptor pair involved. While intracellular delivery may represent an important limiting factor for conjugates targeting intracellular receptors such as NOD1, TLR7, and RIG-I, altered receptor accessibility or steric interference following conjugation may be more relevant determinants of activity for the extracellular TLR4-targeting conjugate **N1/T4**. However, the extent to which altered cellular uptake and receptor accessibility contribute to the observed biological profiles remains unresolved and warrants further investigation. It should also be noted that the difference in expression profile of the relevant PRRs in effector cell populations may also have attributed to the observed limited capacity of conjugates to induce cytolytic activity. NK cells are the primary mediators of cytotoxicity in this assay, yet available evidence suggests that NOD1 and RIG-I expression in human NK cells is relatively low or variable in healthy donors,^25–27^ whereas TLR4 and TLR7 are consistently expressed.^28,29^

Recent studies have highlighted the potential of NOD1 agonists to promote dendritic cell maturation and potentiate CD8^+^ T-cell-mediated responses.^30^ These observations prompted us to extend our investigation beyond *in vitro* systems to determine whether the immunomodulatory potential of these compounds could translate into meaningful biological activity *in vivo*. We therefore evaluated their adjuvant properties in a murine vaccination model using ovalbumin (OVA) as a model antigen. Following a prime-boost vaccination regimen with OVA and **N1**, **N1/T4**, **N1/T7**, and **N1/RI** as the adjuvants (three doses on days 0, 21, 42), the OVA-specific IgG, IgG1, and IgG2a antibody responses were measured. Alum (100 μg) served as a universal benchmark.

The individual NOD1 agonist **N1** induced stronger total IgG responses than unadjuvanted OVA or Alum, in agreement with previous findings reported by Magalhaes et al.^31^ Notably, the NOD1/TLR7 conjugate **N1/T7** elicited a robust systemic immune response, producing significantly higher OVA-specific IgG titers than either OVA alone or the individual NOD1 ligand **N1** (Figure 6). The NOD1/RIG-I conjugate **N1/RI** also enhanced OVA-specific IgG levels relative to unadjuvanted OVA, albeit to a lesser extent than **N1/T7**. In contrast, **N1/T4** was the least active conjugate in this assay, which may be attributed, at least in part, to the species-specific activity of its TLR4 agonist moiety, reported to preferentially activate human rather than murine TLR4.^18^ Antibody isotype analysis further revealed that while **N1/T7** also boosted the OVA-specific IgG1a levels, it preferentially enhanced the anti-OVA IgG2a responses, suggesting induction of a more Th1-biased immune profile. In contrast, the remaining conjugates and the individual NOD1 agonist **N1** did not substantially alter the relative distribution of anti-OVA antibody isotypes. The distinct IgG2a-promoting effect observed for **N1/T7** further supports the notion that covalent conjugation may qualitatively modulate immune polarization beyond simply altering the magnitude of antibody production.

**Figure 6:**
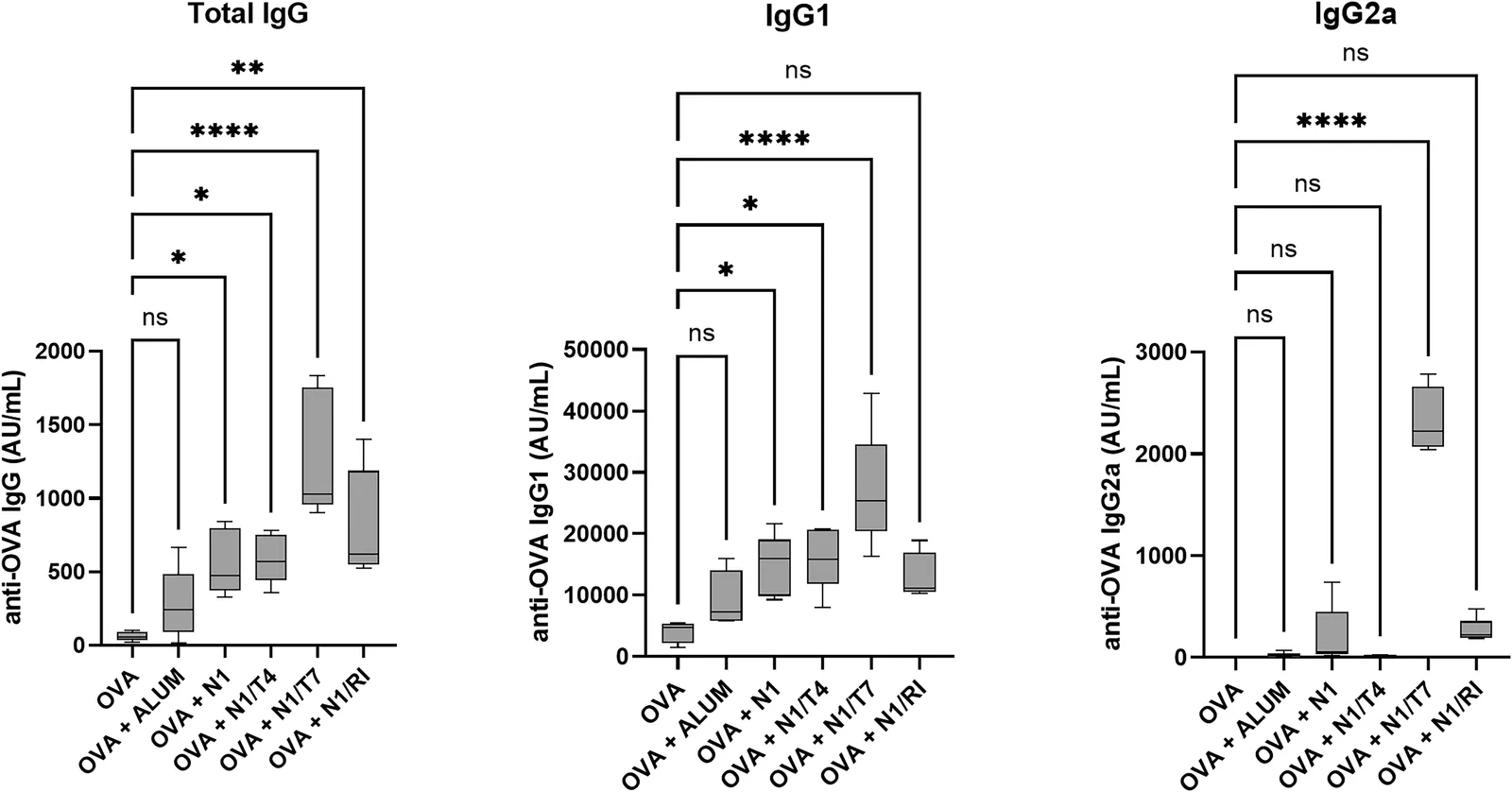
*In vivo* adjuvant activity of conjugated NOD1/PRR agonists. NIH/OlaHsd mice (five per group) were immunized s.c. on days 0, 21, and 42 with OVA alone (10 μg) or OVA (10 μg) and the compounds (**N1/T4**—103 μg, **N1/T7**—79 μg, **N1/RI**—78 μg, and **N1**—50 μg; conjugates’ doses are equimolar to **N1**). OVA-specific IgG, IgG1, and IgG2a responses were determined 7 days after the last dose. *, *p* < 0.05, **, *p* < 0.01, ****, *p* < 0.0001 versus control group (one-way ANOVA posthoc Dunnett’s tests).

Collectively, these findings demonstrate that conjugated NOD1/TLR7 agonist possesses substantial *in vivo* adjuvant activity despite displaying comparatively modest activity in simplified *in vitro* systems. Importantly, the *in vitro* assays employed in the present study were not intended to directly predict antigen-specific humoral immune responses *in vivo*, but rather to provide complementary information regarding innate immune activation and cytotoxic immune signatures. The observed discrepancy therefore likely reflects the fundamentally different biological processes captured by these experimental systems, as well as the greater complexity of immune coordination *in vivo*. Given that TLR7 and NOD1 reside in distinct intracellular compartments, endolysosomal vesicles and cytosol, respectively, dual receptor activation likely involves cellular uptake and intracellular trafficking of the intact conjugate. The predominant uptake mechanism may be context-dependent, with passive diffusion potentially contributing *in vitro*, whereas endocytosis is expected to be more relevant *in vivo*. Following endocytic uptake, TLR7 can be engaged within endolysosomal compartments, whereas access to NOD1 in the cytosol may require endosomal escape and/or intracellular processing of the conjugate. In this context, transport processes associated with SLC15A4, highly expressed in antigen-presenting cells and implicated in endosomal trafficking of NOD1 ligands, may facilitate cytosolic availability of the active moiety.^32^

## Conclusions

In summary, this study demonstrates that covalent conjugation of a NOD1 agonist to structurally distinct PRR agonists generates immunomodulatory profiles that differ substantially from those of the corresponding unconjugated agonists and mixtures. Although conjugation generally attenuated *in vitro* receptor activation and cytokine responses, the conjugates retained pronounced *in vivo* adjuvant activity, most notably **N1/T7**, which additionally promoted a distinct Th1-associated immune profile. These findings highlight the complexity of predicting *in vivo* immunological behavior from simplified *in vitro* systems and support further exploration of NOD1-centered PRR conjugates as modular platforms for next-generation vaccine adjuvant design.

## Experimental Section

### Materials

Chemicals were obtained from Sigma-Aldrich (St. Louis, MO, USA), BLD Pharm (Germany), Acros Organics (Geel, Belgium), ABCR (Germany), Enamine (Monmouth Junction, NJ, USA), and Apollo (Stockport, U.K.), and were used without further purification. Analytical TLC was performed on Merck 60 F254 silica gel plates (0.25 mm), with visualization using ultraviolet light and ninhydrin. Flash column chromatography was carried out on Merck silica gel 60 (particle size 240–400 mesh) and on Biotage Isolera One Flash Chromatograph using Biotage Sfär C18 D Duo 100 Å 30 μm 30 g. 1H and 13C NMR spectra were recorded at 400 and 100 MHz, respectively, on an Avance III spectrometer (Bruker Corporation, Billerica, MA, USA) in MeOD, CDCl_3_ or DMSO-*d*_6_ with tetramethylsilane as the internal standard. Mass spectra were obtained using an Exactive Plus orbitrap mass spectrometer (Thermo Fisher Scientific, Waltham, MA, USA) or on Expression CMS mass spectrometer (Advion Inc., Ithaca, NY, USA). Analytical UHPLC analyses were performed on a Dionex UltiMate 3000 Rapid Separation Binary System (Thermo Fisher Scientific, Waltham, MA, USA) equipped with an autosampler, a binary pump system, a photodiode array detector, a thermostated column compartment, and the Chromeleon Chromatography data system. The column used was Waters Acquity UPLC BEH C18 (1.7 μm, 2.1 × 50 mm), with a flow rate of 0.3 mL/min. The eluent was a mixture of 0.1% TFA in water (A) and acetonitrile (B), with a gradient of (%B): 0–10 min, 5–95%; 10–12 min, 95%; 12–12.5 min, 95–5%. The columns were thermostated at 40 °C. The purity of all biologically tested compounds was >95%.

### General Synthetic Procedures

#### General Procedure A: COMU-Mediated Coupling

To an ice-chilled stirred solution of the Boc-protected amine (1.2 equiv) and carboxylic acid (1 equiv) in DMF, DIPEA (2.5 equiv) and HATU (1.2 equiv) were added. The mixture was allowed to warm to room temperature and the stirring continued overnight, after which it was diluted with DCM and washed with HCl 1 M three times, NaHCO_3_ sat. three times and brine once. Organic phase was then dried over Na_2_SO_4_ and concentrated *in vacuo* to give the desired compounds.

#### General Procedure B: TFA-Mediated Acidolysis

The Boc-protected compound was added to an ice-chilled stirred mixture of TFA and DCM (1:5), and the mixture was allowed to warm to room temperature. After 4 h, the solvent was evaporated *in vacuo*. The residue was washed three times with diethyl ether.

### Characterization of Compounds

#### Diethyl 2-Amino-6-((*tert*-butoxycarbonyl)­amino)­heptanedioate ( **1** )

As reported,^3^ diethyl 2,6-Bis­((*tert*-butoxycarbonyl)­amino)­heptanedioate (1 g, 2.24 mmol) was dissolved in 5 mL of HCl–dioxane and stirred for 2.5 h. The solvent was then removed under vacuum to obtain the hydrochloride salt, which was then dissolved in methanol. Triethylamine (2 equiv) was added until neutralization of the salt, followed by gradual addition of di-*tert*-butyl dicarbonate (490 mg, 2.24 mmol). The reaction mixture was stirred for 30 min on ice, followed by evaporation of the solvent under reduced pressure to obtain the crude product, which was then purified using column chromatography (5% MeOH/CH_2_Cl_2_) to obtain **1** (271 mg, 35%). ^1^H NMR (400 MHz, CDCl_3_) δ = 5.30–5.29 (m, 1H), 4.28–4.16 (m, 5H), 3.96 (s, 1H), 2.04–1.88 (m, 2H), 1.88–1.76 (m, 1H), 1.73–1.62 (m, 1H), 1.62–1.43 (m, 2H), 1.42 (s, 9H), 1.32–1.24 (m, 6H).

#### 10,14-Diethyl 5-Methyl (5*R*)-18,18-dimethyl-3,8,16-trioxo-1-phenyl-2,17-dioxa-4,9,15-triazanonadecane-5,10,14-tricarboxylate ( **2** )

To a stirred solution of (*R*)-4-(((benzyloxy)­carbonyl)­amino)-5-methoxy-5-oxopentanoic acid (767 mg, 2.60 mmol, 1.2 equiv) in dry DMF (5 mL) under an argon atmosphere were added HOBt·H_2_O (351 mg, 2.60 mmol, 1.2 equiv), DIPEA (699 mg, 942 μL, 5.40 mmol, 2.5 equiv), and **1** (750 mg, 2.16 mmol). The reaction mixture was cooled on ice for 30 min, then EDC·HCl (498 mg, 2.60 mmol, 1.2 equiv) and DMAP (13.2 mg, 0.11 mmol, 0.05 equiv) were added. The reaction was then allowed to warm up and the mixture was stirred at room temperature for 16 h. The day after, the reaction was diluted with DCM (50 mL) and washed sequentially with 1 M HCl (3 × 15 mL), saturated NaHCO_3_ (3 × 15 mL), and brine (20 mL). The organic layer was dried over Na_2_SO_4_, filtered, and concentrated under reduced pressure to give the crude protected intermediate, which without any further purification was dissolved in anhydrous MeOH (10 mL) and transferred to a double-neck flask under Ar atmosphere. Pd/C (10 wt %, 75 mg) was added, and the reaction mixture was stirred under H_2_ for 6 h at room temperature. Upon completion, reaction mixture was filtered through a Celite pad, and the filtrate was concentrated under reduced pressure to give **2** as a golden oil (795 mg, 75% over 2 steps). ^1^H NMR (400 MHz, DMSO) δ = 8.22–8.12 (m, 1H), 7.23–7.16 (m, 1H), 4.19–4.12 (m, 1H), 4.10–4.02 (m, 4H), 3.92–3.80 (m, 1H), 3.62 (s, 3H), 2.24–2.13 (m, 2H), 1.87–1.74 (m, 1H), 1.66–1.51 (m, 6H), 1.37 (s, 9H), 1.34–1.29 (m, 3H), 1.23–1.12 (m, 6H).

#### 12-(((Benzyloxy)­carbonyl)­amino)­dodecanoic Acid ( **3** )

To a stirred suspension of 12-aminododecanoic acid (1 g, 5.00 mmol) in 1 M NaHCO_3_ (15 mL, aq.) and dioxane (15 mL) at 0 °C was added dropwise benzyl chloroformate (CbzCl) (730 μL, 5.00 mmol). The reaction mixture was allowed to warm to room temperature and stirred overnight. The day after the reaction mixture was extracted with EtOAc (3 × 30 mL). The combined organic phases were washed with brine, dried over Na_2_SO_4_, filtered, and concentrated under reduced pressure to afford **3** as a white solid (1.54 g, quantitative). ^1^H NMR (400 MHz, DMSO) δ = 7.39–7.27 (m, 5H), 7.22 (t, *J* = 4.6, 1H), 2.96 (q, *J* = 6.6, 2H), 2.51–2.43 (m, 4H), 1.57–1.43 (m, 2H), 1.41–1.34 (m, 2H), 1.27–1.20 (m, 14H).

#### 10,6-Diethyl 15-Methyl (15*R*)-28-amino-2,2-dimethyl-4,12,17-trioxo-3-oxa-5,11,16-triazaoctacosane-6,10,15-tricarboxylate ( **4** )

To a stirred suspension of **3** (837 mg, 2.40 mmol, 1.2 equiv) in dry DMF (5 mL) under an argon atmosphere were added HOBt·H_2_O (324 mg, 2.40 mmol, 1.2 equiv), DIPEA (645 mg, 869 μL, 5.00 mmol, 2.5 equiv), and **2** (795 mg, 1.62 mmol). The reaction mixture was cooled on ice for 30 min, then EDC·HCl (459 mg, 2.40 mmol, 1.2 equiv) and DMAP (12.2 mg, 0.10 mmol, 0.05 equiv) were added. The reaction was allowed to warm to room temperature and stirred for 16 h. The next day, the reaction mixture was diluted with DCM (50 mL) and washed sequentially with 1 M HCl (3 × 15 mL), saturated NaHCO_3_ (3 × 15 mL), and brine (20 mL). The organic phase was dried over Na_2_SO_4_, filtered, and concentrated under reduced pressure to give the crude protected intermediate. The residue was directly dissolved in anhydrous MeOH (10 mL) and transferred to a double-neck flask under argon atmosphere. Pd/C (10 wt %, 79 mg) was added, and the reaction mixture was stirred under H_2_ for 6 h at room temperature. Upon completion, the reaction mixture was filtered through a Celite pad, and the filtrate was concentrated under reduced pressure to give **4** as a transparent oil (590 mg, 50% yield over 2 steps). ^1^H NMR (400 MHz, DMSO) δ = 8.20 (t, 2H), 7.20 (dd, *J* = 7.7, 2.7 Hz, 1H), 4.25–3.98 (m, 6H), 3.90–3.80 (m, 1H), 3.61 (s, 3H), 2.50–2.40 (m, 1H), 2.18 (t, 2H), 2.10 (t, *J* = 7.4 Hz, 1H), 1.92 (t, 2H), 1.83–1.70 (m, 1H), 1.67–1.53 (m, 4H), 1.51–1.43 (m, 2H), 1.37 (s, 9H), 1.35–1.30 (m, 4H), 1.28–1.20 (m, 14H), 1.22–1.12 (m, 6H).

#### 2-(4-(2-(Cyclopentylamino)-1-(*N*-(2,3-dihydro-1*H*-inden-5-yl)-1,5-diphenyl-1*H*-pyrazole-3-carboxamido)-2-oxoethyl)­phenyl)­acetic Acid ( **T4** )

Prepared as reported.^18^ A solution of 5-aminoindane (133 mg, 1 mmol) in dry methanol (2 mL) was added dropwise to a stirred suspension of 1,5-diphenyl-1*H*-pyrazole-3-carboxylic acid (264 mg, 1 mmol), methyl 2-(4 formylphenyl)­acetate (178 mg, 1 mmol) and isocyanocyclopentane (95 mg, 107 μL, 1 mmol) in dry methanol (5 mL) at room temperature. The mixture was stirred at room temperature overnight, then evaporated in vacuo and purified with column chromatography (40–50% gradient of ethyl acetate in hexane). The resulting solid was dissolved in a mixture of tetrahydrofuran (6 mL) and methanol (3 mL), then 1 M sodium hydroxide solution (2.4 mL, 2.4 mmol) was added to give a homogeneous solution. After 3 h at room temperature the mixture was concentrated under reduced pressure to ca. 30% of original volume, diluted with water (8 mL), acidified with 1 M hydrochloric acid to pH = 2, and extracted with ethyl acetate (2 × 20 mL). Combined organic extracts were washed with brine (20 mL), dried over anhydrous magnesium sulfate, filtered, and evaporated in vacuo. The resulting yellow oil was triturated with diethyl ether, the resulting solid filtered, and washed three times with diethyl ether to give compound **T4** as a white powder (257 mg, 40%). ^1^H NMR (400 MHz, DMSO*-d*_6_) δ = 12.28 (s, 1H), 8.10 (d, *J* = 7.0 Hz, 1H), 7.39–7.24 (m, 7H), 7.08 (s, 5H), 7.05–7.00 (m, 3H), 6.97–6.89 (m, 3H), 6.25–6.19 (m, 2H), 4.08–3.98 (m, 1H), 3.47 (s, 2H), 2.74 (t, *J* = 7.4 Hz, 2H), 1.99–1.88 (m, 2H), 1.80–1.73 (m, 2H), 1.68–1.20 (m, 8H).

#### 2-(2-Naphthamido)­benzo­[*d*]­thiazole-6-carboxylic Acid ( **RI** )

Prepared as reported.^17^ To a stirred solution of methyl 2-(2-naphthamido)­benzo­[*d*]­thiazole-6-carboxylate (330 mg, 1.00 mmol) in a 3:1:1 mixture of MeOH/THF/H_2_O (10 mL total; 6 mL MeOH, 2 mL THF, 2 mL H_2_O) was added LiOH·H_2_O (126 mg, 3.00 mmol, 3.0 equiv) at room temperature. The reaction mixture was stirred for 16 h. After completion, the mixture was acidified to pH ≈ 2 using 1 M HCl (≈3 mL, dropwise). The aqueous phase was extracted with EtOAc (3 × 15 mL), and the combined organic layers were washed with brine (15 mL), dried over Na_2_SO_4_, filtered, and concentrated under reduced pressure to afford **RI** as an off-white solid (302 mg, 90%). ^1^H NMR (400 MHz, DMSO-*d*_6_) δ 13.10 (s, 1H), 10.48 (s, 1H), 8.76 (d, J = 8.2 Hz, 1H), 8.19 (d, *J* = 8.6 Hz, 1H), 8.07 (d, *J* = 7.7 Hz, 1H), 7.99 (d, *J* = 8.1 Hz, 1H), 7.89 (t, *J* = 7.8 Hz, 1H), 7.72 (t, *J* = 7.6 Hz, 1H), 7.56 (t, *J* = 7.3 Hz, 1H), 7.45 (t, *J* = 7.6 Hz, 1H), 7.30 (d, *J* = 8.3 Hz, 1H).

#### 4-((6-Amino-2-butoxy-8-hydroxy-9*H*-purin-9-yl)­methyl)­benzoic Acid ( **T7** )

Prepared as reported.^19^ To a solution of 4-((6-amino-8-bromo-2-butoxy-9*H*-purin-9-yl)­methyl)­benzoic acid (1.50 g, 3.6 mmol) in methanol (27 mL) was added sodium hydroxide (28 mL, 10 M, 280 mmol) in a round-bottom flask. The mixture was refluxed for 24 h under stirring. After completion, the solution was cooled to room temperature and transferred to a 250 mL round-bottom flask. Methanol (80 mL) was added, and the mixture was acidified with 6 M HCl (60 mL). The reaction mixture was concentrated under reduced pressure. After cooling in an ice bath, the resulting white precipitate was filtered, washed with cold water, and dried to give **T7** as a white solid (1.43 g, 74%). ^1^H NMR (400 MHz, DMSO-*d*_6_) δ 10.13 (s, 1H), 7.89 (d, *J* = 8.3 Hz, 2H), 7.38 (d, *J* = 8.3 Hz, 2H), 4.93 (s, 2H), 4.14 (t, *J* = 6.6 Hz, 2H), 1.64–1.57 (m, 2H), 1.39–1.31 (m, 2H), 0.89 (t, *J* = 7.4 Hz, 3H).

#### 10,6-Diethyl 15-Methyl (15*R*)-31-(4-(2-(cyclopentylamino)-1-(*N*-(2,3-dihydro-1*H*-inden-5-yl)-1,5-diphenyl-1*H*-pyrazole-3-carboxamido)-2-oxoethyl)­phenyl)-2,2-dimethyl-4,12,17,30-tetraoxo-3-oxa-5,11,16,29-tetraazahentriacontane-6,10,15-tricarboxylate ( **5** )

A solution of **T4** (81.8 mg, 128.1 μmol, 1.1 equiv) in dry DMF (3 mL) was prepared under an argon atmosphere and cooled on ice. To the stirring solution were added HATU (48.7 mg, 128.1 μmol, 1.1 equiv) and DIPEA (51 μL, 291 μmol, 2.5 equiv). After 30 min, **4** (80 mg, 116.5 μmol) and DMAP (2 mg, 16.4 μmol, 0.14 equiv) were added. The reaction mixture was then allowed to warm to room temperature and stirred overnight for 16 h, after which the mixture was diluted with DCM (20 mL) and washed sequentially with 1 M HCl (3 × 10 mL), saturated NaHCO_3_ (3 × 10 mL), and brine (10 mL). The organic layer was dried over anhydrous Na_2_SO_4_, filtered, and concentrated under reduced pressure. The product was purified by reverse-phase column chromatography using a gradient of 5–95% ACN in aqueous 0.1% TFA to afford **5** as a white powder (11.4 mg, 10% yield). ^1^H NMR (400 MHz, MeOD) δ = 8.12–8.01 (m, 1H), 7.36–7.21 (m, 7H), 7.19–7.12 (m, 3H), 7.09–7.02 (m, 2H), 7.00–6.94 (m, 2H), 6.24 (s, 1H), 5.91 (s, 1H), 4.45–4.30 (m, 1H), 4.23–4.10 (m, 4H), 4.09–4.00 (m, 1H), 3.77–3.59 (m, 2H), 3.39 (s, 2H), 3.13 (t, *J* = 7.0 Hz, 2H), 2.82 (t, 2H), 2.38–2.28 (m, 3H), 2.29–2.21 (m, 2H), 2.06–1.74 (m, 7H), 1.73–1.53 (m, 10H), 1.49–1.40 (m, 13H), 1.39–1.23 (m, 27H).

#### Diethyl 2-Amino-6-((4*R*)-4-(12-(2-(4-(2-(cyclopentylamino)-1-(*N*-(2,3-dihydro-1*H*-inden-5-yl)-1,5-diphenyl-1*H*-pyrazole-3-carboxamido)-2-oxoethyl)­phenyl)­acetamido)­dodecanamido)-5-methoxy-5-oxopentanamido)­heptanedioate ( **N1/T4** )

**5** was dissolved in a solution of TFA and DCM (1:5, v/v, 1 mL) and stirred at room temperature for 4 h. The mixture was coevaporated with diethyl ether (3 × 5 mL) and then washed sequentially with saturated NaHCO_3_ (3 × 5 mL), and brine (5 mL). The organic layer was dried over anhydrous Na_2_SO_4_, filtered, and concentrated under reduced pressure to give **N1/T4** as a white powder (quantitative). ^1^H NMR (400 MHz, MeOD) δ = 7.34–7.20 (m, 6H), 7.19–7.09 (m, 4H), 7.09–7.01 (m, 2H), 7.00–6.91 (m, 3H), 6.22 (s, 1H), 5.87 (s, 1H), 4.40 (t, *J* = 6.0 Hz, 2H), 4.28 (q, *J* = 6.7 Hz, 2H), 4.16 (q, *J* = 6.9 Hz, 3H), 4.05–3.97 (m, 1H), 3.69 (s, 3H), 3.38 (s, 2H), 3.12 (t, *J* = 7.0 Hz, 2H), 2.79 (t, *J* = 7.7 Hz, 2H), 2.75–2.70 (m, 2H), 2.37–2.28 (m, 2H), 2.27–2.07 (m, 3H), 2.03–1.79 (m, 8H), 1.74–1.40 (m, 13H), 1.34–1.20 (m, 27H). ^13^C NMR (400 MHz, CDCl_3_/MeOD) δ = 183.38, 174.98, 172.68, 172.04, 171.51, 169.37, 164.21, 146.48, 144.84, 144.17, 144.03, 143.10, 139.30, 138.84, 135.08, 133.72, 130.62, 129.26, 128.80, 128.61, 128.49, 128.07, 126.26, 125.11, 124.07, 109.74, 66.79, 62.67, 61.55, 52.68, 52.60, 52.40, 51.99, 51.85, 51.56, 49.78, 49.57, 49.35, 49.14, 48.93, 48.71, 48.50, 43.07, 39.81, 39.72, 36.13, 32.68, 32.60, 32.49, 29.82, 29.67, 29.39, 29.30, 29.19, 26.77, 25.75, 23.75, 13.99, 13.91. HRMS *m*/*z* calculated for C_69_H_90_N_8_O_11_: 1207.67626 (M + H)^+^, found 1207.67888.

#### 21,25-Diethyl 16-Methyl (*16R*)-1-(4-((6-amino-2-butoxy-8-hydroxy-9*H*-purin-9-yl)­methyl)­phenyl)-29,29-dimethyl-1,14,19,27-tetraoxo-28-oxa-2,15,20,26-tetraazatriacontane-16,21,25-tricarboxylate ( **6** )

A solution of **T7** (45.8 mg, 128.1 μmol, 1.1 equiv) in dry DMF (3 mL) was prepared under an argon atmosphere and cooled on ice. To the stirring solution were added HATU (48.7 mg, 128.1 μmol, 1.1 equiv) and DIPEA (51 μL, 291 μmol, 2.5 equiv). After 30 min, **4** (80 mg, 116.5 μmol) and DMAP (2 mg, 16.4 μmol, 0.14 equiv) were added. The reaction mixture was then allowed to warm to room temperature and stirred overnight for 16 h, after which the mixture was diluted with DCM (20 mL) and washed sequentially with 1 M HCl (3 × 10 mL), saturated NaHCO_3_ (3 × 10 mL), and brine (10 mL). The organic layer was dried over anhydrous Na_2_SO_4_, filtered, and concentrated under reduced pressure. The product was purified by reverse-phase column chromatography using a gradient of 5–95% ACN in aqueous 0.1% TFA to afford **6** as a white powder (13.2 mg, 11% yield). ^1^H NMR (400 MHz, MeOD) δ = 7.76 (d, *J* = 8.3 Hz, 2H), 7.45 (d, *J* = 8.4 Hz, 2H), 5.04 (s, 2H), 4.60 (s, 1H), 4.26 (t, *J* = 6.6 Hz, 2H), 4.11 (s, 4H), 3.71 (s, 3H), 2.36 (s, 2H), 2.25–2.20 (m, 2H), 2.17–2.08 (m, 1H), 1.98–1.89 (m, 1H), 1.86–1.55 (m, 9H), 1.51–1.39 (m, 11H), 1.38–1.22 (m, 22H), 0.95 (t, *J* = 7.4 Hz, 3H).

#### Diethyl 2-Amino-6-((*R*)-4-(12-(4-((6-amino-2-butoxy-8-hydroxy-9*H*-purin-9-yl)­methyl)­benzamido)­dodecanamido)-5-methoxy-5-oxopentanamido)­heptanedioate ( **N1/T7** )

**6** was dissolved in a solution of TFA and DCM (1:5, v/v, 1 mL) and stirred at room temperature for 4 h. The mixture was coevaporated with diethyl ether (3 × 5 mL) and then washed sequentially with saturated NaHCO_3_ (3 × 5 mL), and brine (5 mL). The organic layer was dried over anhydrous Na_2_SO_4_, filtered, and concentrated under reduced pressure to give **N1/T7** as a white powder (quantitative). ^1^H NMR (400 MHz, CDCl_3_/MeOD) δ = 7.65 (d, *J* = 7.7 Hz, 2H), 7.42–7.34 (m, 3H), 5.02–4.93 (m, 3H), 4.41 (s, 2H), 4.32–4.15 (m, 2H), 4.15–4.05 (m, 2H), 3.65 (d, *J* = 2.2 Hz, 3H), 3.11 (s, 1H), 2.30–2.18 (m, 2H), 2.20–2.11 (m, 2H), 1.95–1.79 (m, 3H), 1.74–1.62 (m, 2H), 1.58–1.44 (m, 3H), 1.47–1.30 (m, 3H), 1.14–0.95 (m, 12H), 0.95–0.84 (m, 6H). ^13^C NMR (400 MHz, CDCl_3_/MeOD) δ = 171.11, 168.20, 166.83, 157.68, 127.58, 127.33, 126.41, 75.92, 67.25, 61.73, 60.59, 51.44, 51.04, 50.91, 50.63, 48.90, 48.69, 48.47, 48.26, 48.05, 47.83, 47.62, 42.20, 38.58, 36.45, 36.11, 35.15, 30.94, 29.73, 29.04, 28.70, 28.37, 28.29, 28.15, 26.33, 25.80, 24.67, 21.69, 19.94, 18.72, 18.05, 13.08, 13.01, 12.92, 12.76, 12.72. HRMS *m*/*z* calculated for C_46_H_71_N_9_O_11_: 926.53066 (M + H)^+^, found 926.53458.

#### 21,25-Diethyl 16-Methyl (*16R*)-1-(2-(2-naphthamido)­benzo­[*d*]­thiazol-5-yl)-29,29-dimethyl-1,14,19,27-tetraoxo-28-oxa-2,15,20,26-tetraazatriacontane-16,21,25-tricarboxylate ( **7** )

A solution of **RI** (44.6 mg, 128.1 μmol, 1.1 equiv) in dry DMF (3 mL) was prepared under an argon atmosphere and cooled on ice. To the stirring solution were added HATU (48.7 mg, 128.1 μmol, 1.1 equiv) and DIPEA (51 μL, 291 μmol, 2.5 equiv). After 30 min, **4** (80 mg, 116.5 μmol) and DMAP (2 mg, 16.4 μmol, 0.14 equiv) were added. The reaction mixture was then allowed to warm to room temperature and stirred overnight for 16 h, after which the mixture was diluted with DCM (20 mL) and washed sequentially with 1 M HCl (3 × 10 mL), saturated NaHCO_3_ (3 × 10 mL), and brine (10 mL). The organic layer was dried over anhydrous Na_2_SO_4_, filtered, and concentrated under reduced pressure. The product was purified by reverse-phase column chromatography using a gradient of 5–95% ACN in aqueous 0.1% TFA to afford **7** as a white powder (11.8 mg, 10% yield). ^1^H NMR (400 MHz, CDCl_3_) δ = 8.56 (s, 1H), 8.34 (d, *J* = 1.7 Hz, 1H), 8.07 (d, *J* = 8.7 Hz, 1H), 7.97–7.79 (m, 3H), 6.79–6.63 (m, 1H), 4.76–4.63 (m, 1H), 4.62–4.44 (m, 2H), 3.71 (s, 3H), 3.54–3.41 (m, 2H), 2.37–2.13 (m, 3H), 1.99–1.89 (m, 1H), 1.69–1.54 (m, 6H), 1.45–1.38 (m, 11H), 1.27–1.19 (m, 16H).

#### Diethyl 2-((*R*)-4-(12-(2-(2-Naphthamido)­benzo­[*d*]­thiazole-6-carboxamido)­dodecanamido)-5-methoxy-5-oxopentanamido)-6-aminoheptanedioate ( **N1/RI** )

**7** was dissolved in a solution of TFA and DCM (1:5, v/v, 1 mL) and stirred at room temperature for 4 h. The mixture was coevaporated with diethyl ether (3 × 5 mL) and then washed sequentially with saturated NaHCO_3_ (3 × 5 mL), and brine (5 mL). The organic layer was dried over anhydrous Na_2_SO_4_, filtered, and concentrated under reduced pressure to give **N1/RI** as a white powder (quantitative). ^1^H NMR (400 MHz, CDCl_3_/MeOD) δ = 8.64–8.54 (m, 1H), 8.52 (d, *J* = 2.0 Hz, 1H), 8.29–8.24 (m, 1H), 8.12–8.01 (m, 2H), 7.95 (t, *J* = 7.7 Hz, 3H), 7.87 (d, *J* = 8.0 Hz, 2H), 7.84–7.78 (m, 1H), 7.77–7.70 (m, 1H), 7.63–7.50 (m, 2H), 7.00 (t, *J* = 5.7 Hz, 1H), 4.48–4.32 (m, 2H), 4.21–4.04 (m, 4H), 3.90 (s, 3H), 3.66 (s, 3H), 3.44–3.35 (m, 1H), 3.34–3.28 (m, 1H), 2.37–2.19 (m, 2H), 2.20–2.11 (m, 2H), 2.00–1.86 (m, 1H), 1.89–1.74 (m, 2H), 1.69–1.46 (m, 6H), 1.42–1.23 (m, 10H), 0.92–0.71 (m, 6H). ^13^C NMR (400 MHz, CDCl_3_/MeOD) δ = 172.34, 167.12, 166.39, 151.82, 135.66, 132.64, 129.47, 129.43, 129.04, 129.01, 128.83, 127.98, 127.84, 127.30, 125.42, 123.86, 123.78, 120.97, 120.42, 120.33, 77.44, 61.60, 52.50, 52.40, 52.27, 52.20, 49.93, 49.72, 49.50, 49.29, 49.08, 48.86, 48.65, 40.36, 38.99, 37.18, 36.28, 33.80, 32.84, 32.01, 30.23, 30.11, 29.81, 29.74, 29.57, 29.49, 29.33, 29.27, 27.51, 27.17, 27.06, 26.83, 26.01, 25.72, 22.77, 21.40, 19.78, 19.27, 14.14, 14.12, 10.94. HRMS *m*/*z* calculated for C_48_H_64_N_6_O_10_S: 917.44382 (M + H)^+^, found 917.44774.

### HEK-Blue NOD1, TLR4, TLR7 Cells and HEK-Lucia RIG-I Cells

HEK-Blue NOD1 (Cat. code: hkb-hnod1), TLR4 (Cat. code: hkb-htlr4), and TLR7 (Cat. code: hkb-htlr7) cell lines (Invivogen, San Diego, CA) are derived from HEK293 cells by cotransfection of hNOD1, hTLR4 or hTLR7 genes, respectively, and a NF-κB-inducible SEAP reporter gene. Following activation of the respective receptors, the resulting NF-κB induces the production of SEAP, the levels of which can be quantified colorimetrically. HEK-Lucia RIG-I cells (Cat. code: hkl-hrigi, Invivogen, San Diego, CA) are derived from HEK293 cells by cotransfection of hRIG-I gene and an IFN-inducible Lucia luciferase reporter gene. Following activation, the resulting IFN induces the Lucia luciferase activity, which can be quantified colorimetrically. HEK-Blue cells and HEK-Lucia cells were cultured according to the manufacturer instructions, in Dulbecco’s modified Eagle’s medium (Sigma-Aldrich, St. Louis, MO) supplemented with 10% heat-inactivated fetal bovine serum (Gibco), 2 mM l-glutamine (Sigma-Aldrich), 100 U/mL penicillin (Sigma-Aldrich), 100 μg/mL streptomycin (Sigma-Aldrich), and 100 μg/mL Normocin (Invivogen) for two passages. All of the subsequent passages were cultured in medium additionally supplemented with 100 μg/mL Zeocin (Invivogen) and 30 μg/mL Blasticidin (Invivogen) for HEK-Blue hNOD1 cells and HEK-Lucia RIG-I cells, with 100 μg/mL Zeocin (Invivogen) and 10 μg/mL Blasticidin (Invivogen) for HEK-Blue hTLR7, or 1× HEK-Blue Selection for HEK-Blue hTLR4 cells. The cells were incubated in a humidified atmosphere at 37 °C and 5% CO_2_.

### Peripheral Blood Mononuclear Cells

Human PBMCs from healthy and consenting donors were isolated from heparinized blood by density gradient centrifugation with Ficoll-Paque (Pharmacia, Sweden). The isolated cells were washed twice with PBS, resuspended in RPMI 1640 medium (Sigma-Aldrich, St. Louis, MO, USA) supplemented with 10% heat-inactivated fetal bovine serum (Gibco), 2 mM l-glutamine (Sigma-Aldrich), 100 U/mL penicillin (Sigma-Aldrich), and 100 μg/mL streptomycin (Sigma-Aldrich), and used in the assays.

### Cytotoxicity

The tested compounds were dissolved in DMSO and further diluted in culture medium to the desired final concentrations, such that the final DMSO concentration never exceeded 0.1%. HEK-Blue hNOD1, hTLR4, hTLR7 cells, and HEK-Lucia RIG-I cells were seeded (40,000 cells/well) in 96-well plates in 100 μL of culture medium, and treated with 5, 10, 20, or 100 μM of each compound (depending on the highest concentration used for the ensuing reporter assays) or with the corresponding vehicle (0.1% DMSO; control cells). After 18 h of incubation (37 °C, 5% CO_2_), the metabolic activity was assessed using the CellTiter 96 Aqueous One Solution cell proliferation assay (Promega, Madison, WI, USA), according to the manufacturer instructions. The experiments were run in duplicates, and repeated as two independent biological replicates. Statistical significance was determined with one-way ANOVA with subsequent Dunnett’s multiple comparisons test.

### Measurement of NF-κB Transcriptional Activity in HEK-Blue Cells

HEK-Blue hNOD1, hTLR4 or hTLR7 cells were seeded (25,000 cells/well) in duplicate in 96-well plates in 100 μL of HEK-Blue detection medium (Invivogen, San Diego, CA) and treated in duplicates with the compounds (for assessment of dose response, three or four different concentrations ranging from 5 nM to 20 μM for hNOD1 cells, 0.5 μM to 5 μM for hTLR4 cells, or 10 nM to 25 μM for hTLR7 cells) or with the corresponding vehicle (0.1% DMSO). After 18 h of incubation (37 °C, 5% CO_2_), SEAP activity was determined spectrophotometrically as absorbance at 630 nm (BioTek Synergy microplate reader; Winooski, VT).

### Measurement of IRF-Induced Lucia Luciferase Activity in HEK-Lucia Cells

HEK-Lucia RIG-I cells were seeded (50,000 cells/well) in 96-well plates in 100 μL of growth medium without added selection antibiotics and treated in duplicate with the with the compounds (three different concentrations ranging from 1 μM to 100 μM for assessment of dose response) or with the corresponding vehicle (0.1% DMSO). After 18 h of incubation (37 °C, 5% CO_2_), 10 μL of supernatants were transferred to 96-well white opaque plates. 50 μL of Quanti-Luc 4 detection medium (Invivogen, San Diego, CA) was added and the activity of IRF-induced Lucia luciferase was immediately determined luminometrically (BioTek Synergy microplate reader; Winooski, VT).

### Cytokine Release from Peripheral Blood Mononuclear Cells

Peripheral blood mononuclear cells were seeded (1 × 10^6^ cells/mL) in 96-well plates in 100 μL of growth medium and treated with conjugated agonists (1 and 10 μM), individual agonists (1 and 10 μM), unlinked mixtures of agonists (1 and 10 μM), or the corresponding vehicle (0.1% DMSO). Cell-free supernatants were collected after 18 h of incubation (37 °C, 5% CO_2_) and stored at −80 °C until tested. Cytokine concentrations were determined with the LEGENDplex HU Essential Immune Response Panel (Biolegend, San Diego, CA) (comprising IL-4, IL-2, CXCL10 (IP-10), IL-1β, TNF-α, CCL2 (MCP-1), IL-17A, IL-6, IL-10, IFN-γ, IL-12p70, CXCL8 (IL-8), TGF-β1) on an Attune NxT flow cytometer (Thermo Fisher Scientific, Waltham, MA). Standard curves were generated using recombinant cytokines contained in the kit. The data were analyzed using the FlowJo (Tree Star, Inc., Ashland, OR) and Prism (GraphPad, San Diego, CA) software. Statistical significance was determined with one-way ANOVA with subsequent Dunnett’s multiple comparisons test.

### Peripheral Blood Mononuclear Cell Cytotoxicity Assay

PBMCs were seeded (4 × 10^5^ cells/well) in duplicate in 96-well U-bottom plates and treated with conjugated agonists (1 and 10 μM), unlinked mixtures of agonists (1 and 10 μM), control (IL-2, 200 U/mL) or vehicle (0.1% DMSO) for 18 h. K562 cells were stained with CFSE (Invitrogen, Carlsbad, CA), washed twice with complete medium, and added (1 × 10^4^ cells/well) to the pretreated PBMCs for a final effector cell to target tumor cell ratio of 40:1. After a 4 h coincubation (37 °C, 5% CO_2_), the cells were stained with Sytox blue dead cell stain (Invitrogen) and analyzed using an Attune NxT flow cytometer (Thermo Fisher Scientific, Waltham, MA) and the FlowJo software (Tree Star, Inc., Ashland, OR). Cells that were positive for both CFSE and Sytox blue were defined as dead K562 cells. PBMCs alone and CFSE-labeled cancer cells alone were also treated with the compounds at the same concentrations and stained with Sytox blue to exclude any direct cytotoxicity of the compounds toward the PBMCs and cancer cells. Statistical significance was determined using one-way ANOVA with posthoc Bonferroni’s test.

### In Vivo Vaccination Model

Female NIH/OlaHsd inbred mice (2.0–2.5 months old) were bred at the Institute of Immunology, Croatia. Throughout the study, animals were maintained in the institute’s Animal Facility under standard conditions with ad libitum access to food and water. All procedures involving animals were conducted in accordance with the Croatian Animal Welfare Act (2017) and were fully compliant with EC Directive 2010/63/EU. The study protocol was approved by the Ethical Committee of the Institute of Immunology and by the Directorate of Veterinary and Food Safety of the Ministry of Agriculture of the Republic of Croatia (approval No. HR-POK-009). Sex-matched NIH/OlaHsd mice (*n* = 5 per group) were immunized subcutaneously at the base of the tail on days 0, 21, and 42 with either OVA (10 μg; Serva, Germany) alone or in combination with the tested compounds (**N1/T4**—103 μg, **N1/T7**—79 μg, **N1/RI**—78 μg, and **N1**—50 μg, with conjugate doses adjusted to be equimolar relative to **N1**) and Al-hydrogel (100 μg; Invivogen, San Diego, CA). Each animal received a total injection volume of 0.1 mL. Seven days after administration of the second booster dose, mice were anesthetized by intraperitoneal injection of ketamine/xylazine (25 mg/kg each), followed by blood collection from the axillary plexus. Serum samples were obtained individually, heat-inactivated at 56 °C for 35 min to eliminate complement activity, and stored at −20 °C until further analysis.

### Measurement of Ovalbumin-Specific Serum Antibody Concentration

The concentrations of OVA-specific total IgG, IgG1, and IgG2a antibodies in mouse sera were determined by ELISA. High-binding ELISA plates (Costar) were coated overnight with OVA (1.5 mg/mL; Serva, Germany) prepared in carbonate buffer (pH 9.6). To minimize nonspecific binding, the plates were subsequently blocked for 2 h at 37 °C with 0.5% (w/v) bovine serum albumin in PBS-T (0.05% [v/v] Tween 20 in PBS). After washing, five serial dilutions of mouse serum samples and corresponding standards were applied in duplicate. The plates were incubated overnight at room temperature. Following additional washing steps, bound antibodies were detected using HRP-conjugated goat antimouse IgG (Bio-Rad Laboratories), incubated for 2 h at 37 °C. The enzymatic reaction was visualized by adding a substrate solution containing 0.6 mg/mL *o*-phenylenediamine dihydrochloride in citrate-phosphate buffer (pH 5.0) supplemented with 0.5 μL/mL of 30% H_2_O_2_. After incubation for 30 min at room temperature in the dark, the reaction was stopped with 12.5% H_2_SO_4_, and absorbance was measured at 492 nm using a microplate reader (Thermo Fisher Scientific, Waltham, MA). For OVA-specific IgG1 and IgG2a detection, plates were incubated with biotin-conjugated rat antimouse IgG1 or IgG2a (PharMingen, Becton Dickinson) for 2 h at 37 °C, followed by incubation with streptavidin-peroxidase (Pharmingen) under the same conditions. After washing, the substrate solution was added and incubated for 30 min at room temperature in the dark. The reaction was terminated with 12.5% H_2_SO_4_, and absorbance at 492 nm was recorded. Antibody concentrations were calculated using parallel line analysis with standard preparations of anti-OVA IgG (20,000 AU/mL), anti-OVA IgG1 (400,000 AU/mL), and anti-OVA IgG2a (5000 AU/mL). Statistical significance between groups was evaluated by one-way ANOVA followed by Dunnett’s multiple comparisons test.

### Statistics

All the experiments were performed at least two times, with average values expressed as means ± standard error of mean (SEM). The data were analyzed using Prism software (version 10; GraphPad Software, CA, USA). Statistical significance was determined according to the specific procedures outlined in each experiment. Differences were considered significant (*) for *P* < 0.05, highly significant (**) for *P* < 0.01, and extremely significant for *P* < 0.001 and *P* < 0.0001 (*** and ****, respectively).

## Supplementary Material



## Notes

1H and 13C NMR spectra of final compounds; HPLC traces of final compounds; HRMS spectra of final compounds; Supporting Figures (PDF)

## Abbreviations

Boc: tert-butoxycarbonyl
Cbz: benzyloxycarbonyl
CARD: caspase activation and recruitment domain
COMU: (1-cyano-2-ethoxy-2-oxoethylidenaminooxy)­dimethylamino-morpholino-carbenium hexafluorophosphate
C12-iE-DAP: *N*-dodecanoyl-γ-d-glutamyl-meso-diaminopimelic acid
DAP: diaminopimelic acid
DCM: dichloromethane
DIPEA: *N*,*N*-diisopropylethylamine
DMAP: 4-dimethylaminopyridine
DMF: dimethylformamide
DMSO: dimethyl sulfoxide
EDC: 1-ethyl-3-(3-(dimethylamino)­propyl)­carbodiimide
GM-CSF: granulocyte–macrophage colony-stimulating factor
HATU: hexafluorophosphate azabenzotriazole tetramethyl uronium
HEK: human embryonic kidney
HOBt: 1-hydroxybenzotriazole
IFN-γ: interferon-γ
IL: interleukin
IP-10: interferon-γ-induced protein 10
IRF7: interferon regulatory factor 7
JNK: c-Jun N-terminal kinase
LPS: lipopolysaccharide
MAPK: mitogen-activated protein kinase
MAVS: mitochondrial antiviral-signaling protein
MDA5: melanoma differentiation-associated protein 5
MCP-1: monocyte chemoattractant protein-1
MTS: (3-(4,5-dimethylthiazol-2-yl)-5-(3-carboxymethoxyphenyl)-2-(4-sulfophenyl)-2*H*-tetrazolium) assay
NF-κB: nuclear factor κB
NK: natural killer
NLR: nucleotide-binding oligomerization domain-like receptors
NOD1: nucleotide-binding oligomerization domain-containing protein 1
NOD2: nucleotide-binding oligomerization domain-containing protein 2
PBMC: peripheral blood mononuclear cells
PRR: pattern-recognition receptor
RIG-I: retinoic acid-inducible gene I
RLR: RIG-I-like receptor
SEAP: secreted embryonic alkaline phosphatase
SEM: standard error of the mean
TFA: trifluoroacetic acid
TLR: toll-like receptor
TNF-α: tumor necrosis factor-α.

## References

[1] Lavelle E. C., Murphy C., O’Neill L. A.
J., Creagh E. M. (2010). The Role
of TLRs, NLRs, and RLRs in Mucosal Innate Immunity and Homeostasis. Mucosal Immunol..

[2] Kawai T., Akira S. (2011). Toll-like Receptors
and Their Crosstalk with Other Innate Receptors
in Infection and Immunity. Immunity.

[3] Agnihotri G., Ukani R., Malladi S. S., Warshakoon H. J., Balakrishna R., Wang X., David S. A. (2011). Structure–Activity
Relationships in Nucleotide Oligomerization Domain 1 (Nod1) Agonistic
γ-Glutamyldiaminopimelic Acid Derivatives. J. Med. Chem..

[4] Vegna S., Gregoire D., Moreau M., Lassus P., Durantel D., Assenat E., Hibner U., Simonin Y. (2016). NOD1 Participates in
the Innate Immune Response Triggered by Hepatitis C Virus Polymerase. J. Virol..

[5] Fritz J. H., Girardin S. E., Fitting C., Werts C., Mengin-Lecreulx D., Caroff M., Cavaillon J.-M., Philpott D. J., Adib-Conquy M. (2005). Synergistic
Stimulation of Human Monocytes and Dendritic Cells by Toll-like Receptor
4 and NOD1- and NOD2-Activating Agonists. Eur.
J. Immunol..

[6] Tada H., Aiba S., Shibata K.-I., Ohteki T., Takada H. (2005). Synergistic
Effect of Nod1 and Nod2 Agonists with Toll-Like Receptor Agonists
on Human Dendritic Cells To Generate Interleukin-12 and T Helper Type
1 Cells. Infect. Immun..

[7] Li D., Wu M. (2021). Pattern Recognition
Receptors in Health and Diseases. Signal Transduction
Targeted Ther..

[8] van
Heel D. A., Ghosh S., Butler M., Hunt K., Foxwell B. M. J., Mengin-Lecreulx D., Playford R. J. (2005). Synergistic Enhancement
of Toll-like Receptor Responses by NOD1 Activation. Eur. J. Immunol..

[9] Tukhvatulin A. I., Gitlin I. I., Shcheblyakov D. V., Artemicheva N. M., Burdelya L. G., Shmarov M. M., Naroditsky B. S., Gudkov A. V., Gintsburg A. L., Logunov D. Y. (2013). Combined Stimulation
of Toll-Like Receptor 5 and NOD1 Strongly Potentiates Activity of
NF-κB, Resulting in Enhanced Innate Immune Reactions and Resistance
to Salmonella Enterica Serovar Typhimurium Infection. Infect. Immun..

[10] Wu X. M., Zhang J., Li P. W., Hu Y. W., Cao L., Ouyang S., Bi Y. H., Nie P., Chang M. X. (2020). NOD1 Promotes
Antiviral Signaling by Binding Viral RNA and Regulating the Interaction
of MDA5 and MAVS. J. Immunol..

[11] Guzelj S., Jakopin Ž. (2022). Nucleotide-Binding Oligomerization Domain 1/Toll-Like
Receptor 4 Co-Engagement Promotes Non-Specific Immune Response Against
K562 Cancer Cells. Front. Pharmacol..

[12] Budikhina A. S., Murugina N. E., Maximchik P. V., Dagil Y. A., Nikolaeva A. M., Balyasova L. S., Murugin V. V., Selezneva E. M., Pashchenkova Y. G., Chkadua G. Z., Pinegin B. V., Pashenkov M. V. (2021). Interplay
between NOD1 and TLR4 Receptors in Macrophages: Nonsynergistic Activation
of Signaling Pathways Results in Synergistic Induction of Proinflammatory
Gene Expression. J. Immunol..

[13] Schwarz H., Posselt G., Wurm P., Ulbing M., Duschl A., Horejs-Hoeck J. (2013). TLR8 and NOD
Signaling Synergistically Induce the Production
of IL-1β and IL-23 in Monocyte-Derived DCs and Enhance the Expression
of the Feedback Inhibitor SOCS2. Immunobiology.

[14] Tom J. K., Albin T. J., Manna S., Moser B. A., Steinhardt R. C., Esser-Kahn A. P. (2019). Applications of Immunomodulatory Immune Synergies to
Adjuvant Discovery and Vaccine Development. Trends Biotechnol..

[15] Guzelj S., Weiss M., Slütter B., Frkanec R., Jakopin Ž. (2022). Covalently
Conjugated NOD2/TLR7 Agonists Are Potent and Versatile Immune Potentiators. J. Med. Chem..

[16] Janež Š., Guzelj S., Jakopin Ž. (2024). Linker
Chemistry and Connectivity
Fine-Tune the Immune Response and Kinetic Solubility of Conjugated
NOD2/TLR7 Agonists. Bioconjugate Chem..

[17] Bedard, K. ; Goldberg, D. R. ; Probst, P. Activators of the Retinoic Acid Inducible Gene ″RIG-1″ pathway and Methods of Use Thereof. WO Patent WO033782A12020A1, 2020.

[18] Marshall J. D., Heeke D. S., Rao E., Maynard S. K., Hornigold D., McCrae C., Fraser N., Tovchigrechko A., Yu L., Williams N., King S., Cooper M. E., Hajjar A. M., Woo J. C. (2016). A Novel Class of
Small Molecule Agonists with Preference
for Human over Mouse TLR4 Activation. PLoS One.

[19] Akinbobuyi B., Byrd M. R., Chang C. A., Nguyen M., Seifert Z. J., Flamar A.-L., Zurawski G., Upchurch K. C., Oh S., Dempsey S. H., Enke T. J., Le J., Winstead H. J., Boquín J. R., Kane R. R. (2015). Facile Syntheses of Functionalized
Toll-like Receptor 7 Agonists. Tetrahedron Lett..

[20] Bargh J. D., Isidro-Llobet A., Parker J. S., Spring D. R. (2019). Cleavable Linkers
in Antibody–Drug Conjugates. Chem. Soc.
Rev..

[21] Balamkundu S., Liu C.-F. (2023). Lysosomal-Cleavable Peptide Linkers in Antibody–Drug
Conjugates. Biomedicines.

[22] Su D., Zhang D. (2021). Linker Design Impacts
Antibody-Drug Conjugate Pharmacokinetics and
Efficacy via Modulating the Stability and Payload Release Efficiency. Front. Pharmacol..

[23] Janež Š., Guzelj S., Šišić M., Frkanec R., Pajk S., Burgmeijer L., Slütter B., Jakopin Ž. (2026). Probing Immune Signatures of Conjugated
Pattern Recognition
Receptor Ligands Identifies Chimeras with Adjuvant and Antitumor Activities. J. Med. Chem..

[24] Liu T., Long X., Zhang Y., Jin J., Chen L., Liu A. (2022). IP-10 Enhances
the Amplification Capacity and Antitumor Activity
of CAR-T Cells *in Vitro* and Could Influence Positive
Outcomes in MM Patients Treated with CAR-T Cell Therapy. Int. Immunopharmacol..

[25] Qiu F., Maniar A., Diaz M. Q., Chapoval A. I., Medvedev A. E. (2011). Activation
of Cytokine-Producing and Antitumor Activities of Natural Killer Cells
and Macrophages by Engagement of Toll-like and NOD-like Receptors. Innate Immun..

[26] Knelson E. H., Ivanova E. V., Tarannum M., Campisi M., Lizotte P. H., Booker M. A., Ozgenc I., Noureddine M., Meisenheimer B., Chen M., Piel B., Spicer N., Obua B., Messier C. M., Shannon E., Mahadevan N. R., Tani T., Schol P. J., Lee-Hassett A. M., Zlota A., Vo H. V., Ha M., Bertram A. A., Han S., Thai T. C., Gustafson C. E., Venugopal K., Haggerty T. J., Albertson T. P., Hartley A.-V., Eser P. O., Li Z.-H., Cañadas I., Vivero M., De Rienzo A., Richards W. G., Abu-Yousif A. O., Appleman V. A., Gregory R. C., Parent A., Lineberry N., Smith E. L., Jänne P. A., Miret J. J., Tolstorukov M. Y., Romee R., Paweletz C. P., Bueno R., Barbie D. A. (2022). Activation of Tumor-Cell STING Primes
NK-Cell Therapy. Cancer Immunol. Res..

[27] Girone C., Calati F., Lo Cigno I., Salvi V., Tassinari V., Schioppa T., Borgogna C., Severini L. L., Hiscott J., Cerboni C., Soriani A., Bosisio D., Gariglio M. (2023). The RIG-I
Agonist M8 Triggers Cell Death and Natural Killer Cell Activation
in Human Papillomavirus-Associated Cancer and Potentiates Cisplatin
Cytotoxicity. Cancer Immunol. Immunother..

[28] Lombardi V., Van Overtvelt L., Horiot S., Moingeon P. (2009). Human Dendritic Cells
Stimulated via TLR7 and/or TLR8 Induce the Sequential Production of
IL-10, IFN-γ, and IL-17A by Naive CD4+ T Cells. J. Immunol..

[29] Wen C.-J., Chang C.-H., Chen C.-Y., Peng J.-K., Huang H.-L., Chuang P.-N., Chen C.-Y., Tsai J.-S. (2021). Age-Dependent Messenger
RNA Expression of Toll-like Receptor 4 and Intercellular Adhesion
Molecule-1 in Peripheral Blood Mononuclear Cells. Eur. J. Clin. Invest..

[30] Zhang F., Jiang Q., Cai J., Meng F., Tang W., Liu Z., Lin X., Liu W., Zhou Y., Shen X., Xue R., Dong L., Zhang S. (2024). Activation of NOD1 on Tumor-Associated
Macrophages Augments CD8+ T Cell–Mediated Antitumor Immunity
in Hepatocellular Carcinoma. Sci. Adv..

[31] Magalhaes J. G., Rubino S. J., Travassos L. H., Le Bourhis L., Duan W., Sellge G., Geddes K., Reardon C., Lechmann M., Carneiro L. A., Selvanantham T., Fritz J. H., Taylor B. C., Artis D., Mak T. W., Comeau M. R., Croft M., Girardin S. E., Philpott D. J. (2011). Nucleotide
Oligomerization Domain-Containing Proteins Instruct T Cell Helper
Type 2 Immunity Through Stromal Activation. Proc. Natl. Acad. Sci. U.S.A..

[32] Bharadwaj R., Jaiswal S., Silverman N. (2024). Cytosolic
delivery of innate immune
agonists. Trends Immunol.

