# Supplementary material for: Conjugated NOD1-Centered Dual Agonists Exhibit Modest In Vitro Immunomodulation but Potent In Vivo Adjuvanticity
Source: ACS Bio Med Chem Au. 2026 Jun 15;6(4):410–22. doi: 10.1021/acsbiomedchemau.6c00080 (PMC13495014; doi:10.1021/acsbiomedchemau.6c00080)
Supplement: Supplementary file 1 [file bg6c00080_si_001.pdf]

SUPPORTING INFORMATION

Conjugated NOD1-Centered Dual Agonists Exhibit Modest In Vitro Immunomodulation but Potent In Vivo Adjuvanticity

Emiliano Paradiso,[1] Špela Janež,[1] Veronika Weiss,[1] Ruža Frkanec,[2] Žiga Jakopin*,[1]

[1]Faculty of Pharmacy, University of Ljubljana, SI-1000 Ljubljana, Slovenia

[2]Centre for Research and Knowledge Transfer in Biotechnology, University of Zagreb, 10000 Zagreb, Croatia

*Corresponding Author

Žiga Jakopin

Phone: +386 1 4769 646

Fax: +386 1 4258 031

E-mail: ziga.jakopin@ffa.uni-lj.si

Table of Contents

1. NMR spectra of final compounds 2

2. UHPLC traces of final compounds 8

3. HRMS of final compounds 11

4. Supporting Figures 14

1. NMR spectra of final compounds

Compound N1/T4: ^{1}H, 400 MHz, MeOD

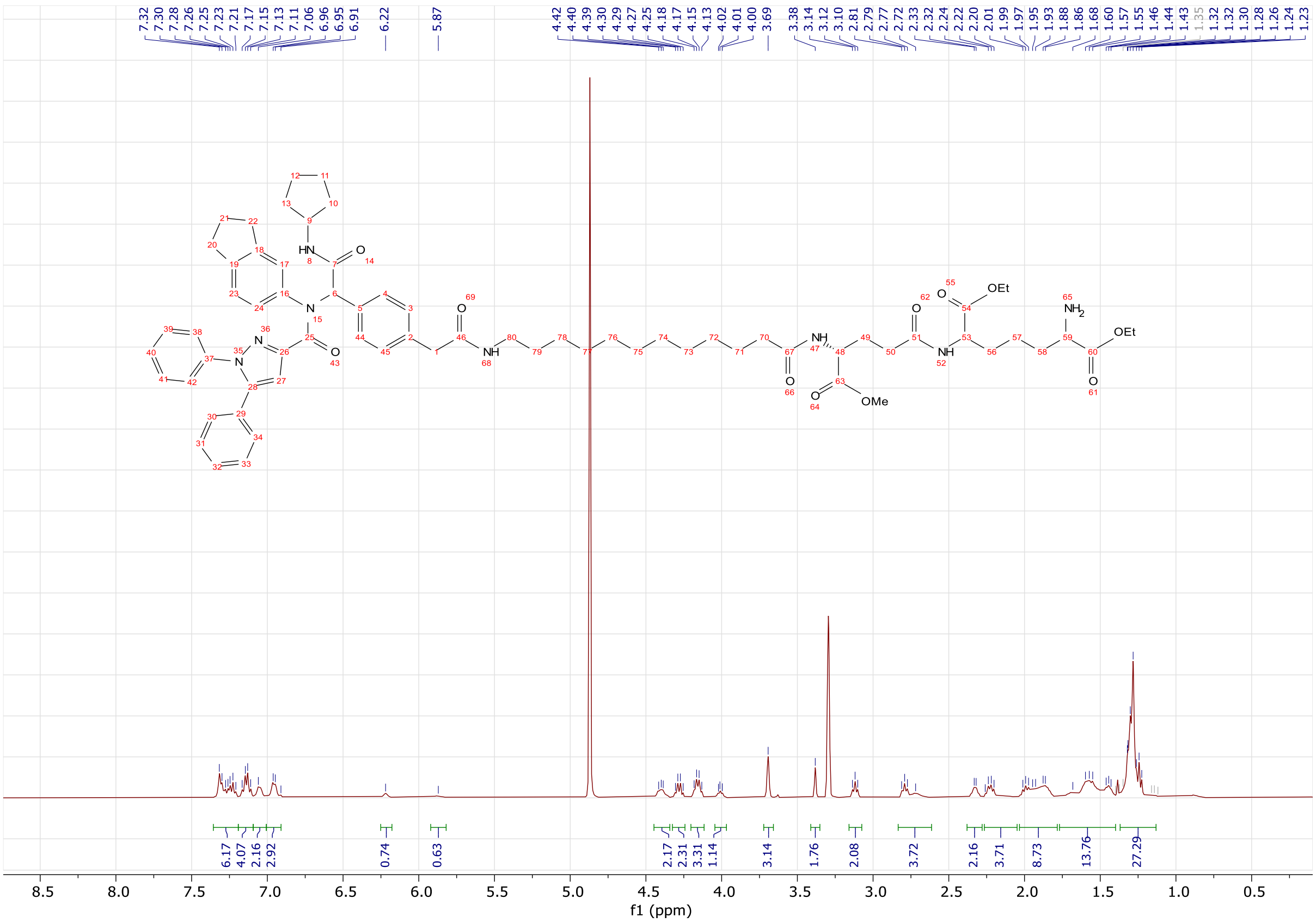

Compound N1/T4: ^{13}C, 400 MHz, $CDCl_3$/MeOD

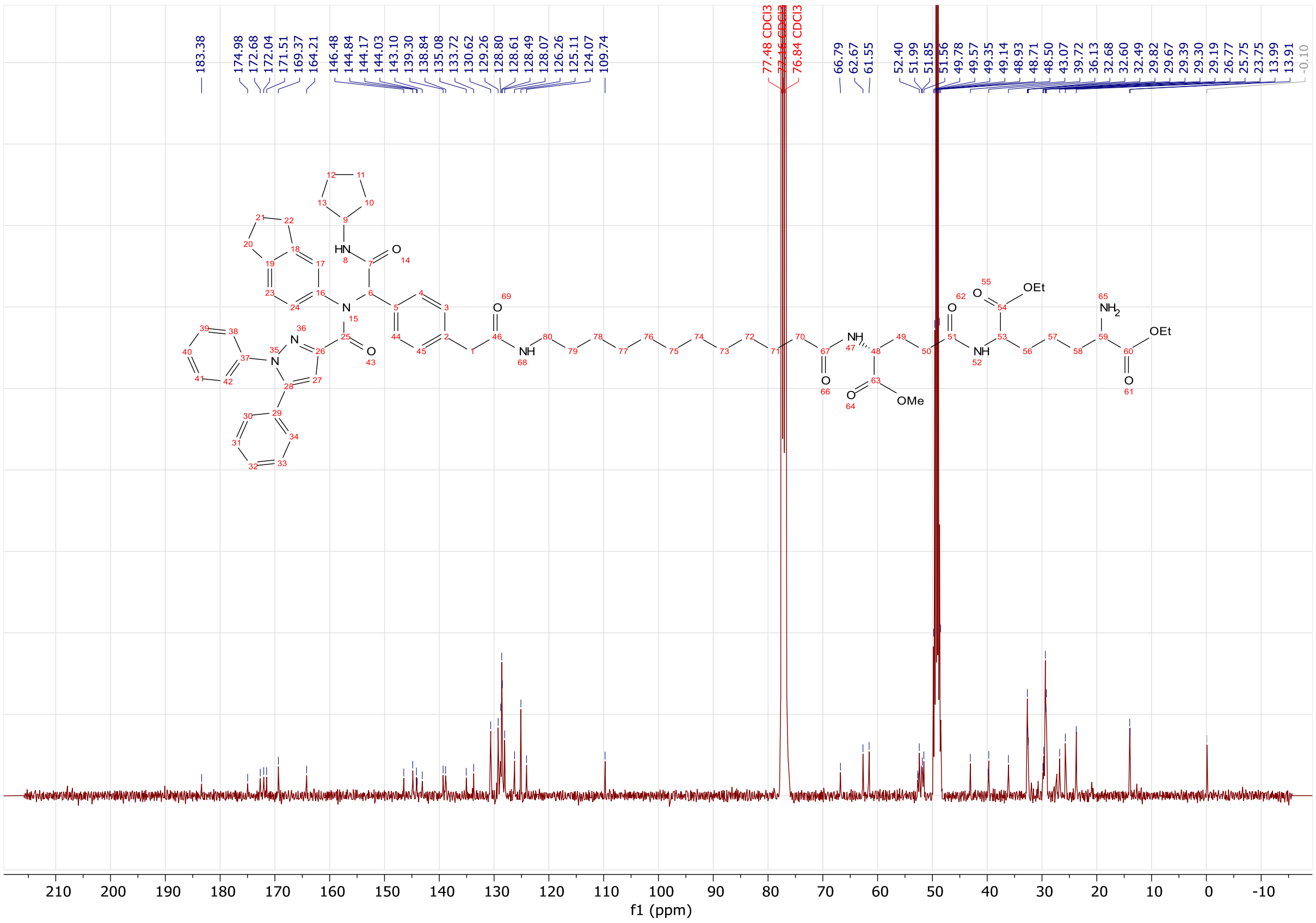

Compound N1/RI: ^{1}H, 400 MHz, $CDCl_3$/MeOD

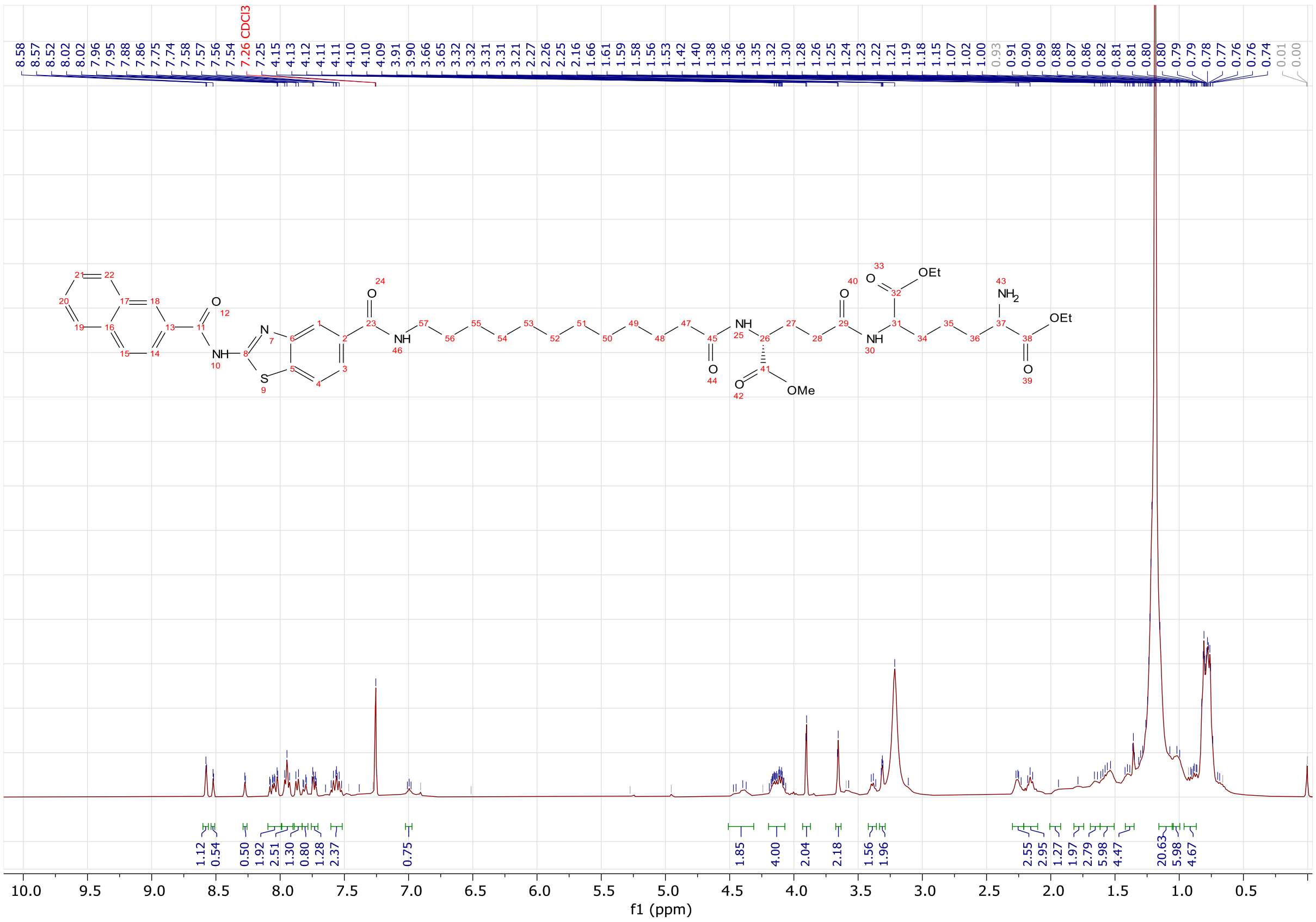

Compound N1/RI: ^{13}C, 400 MHz, $CDCl_3$/MeOD

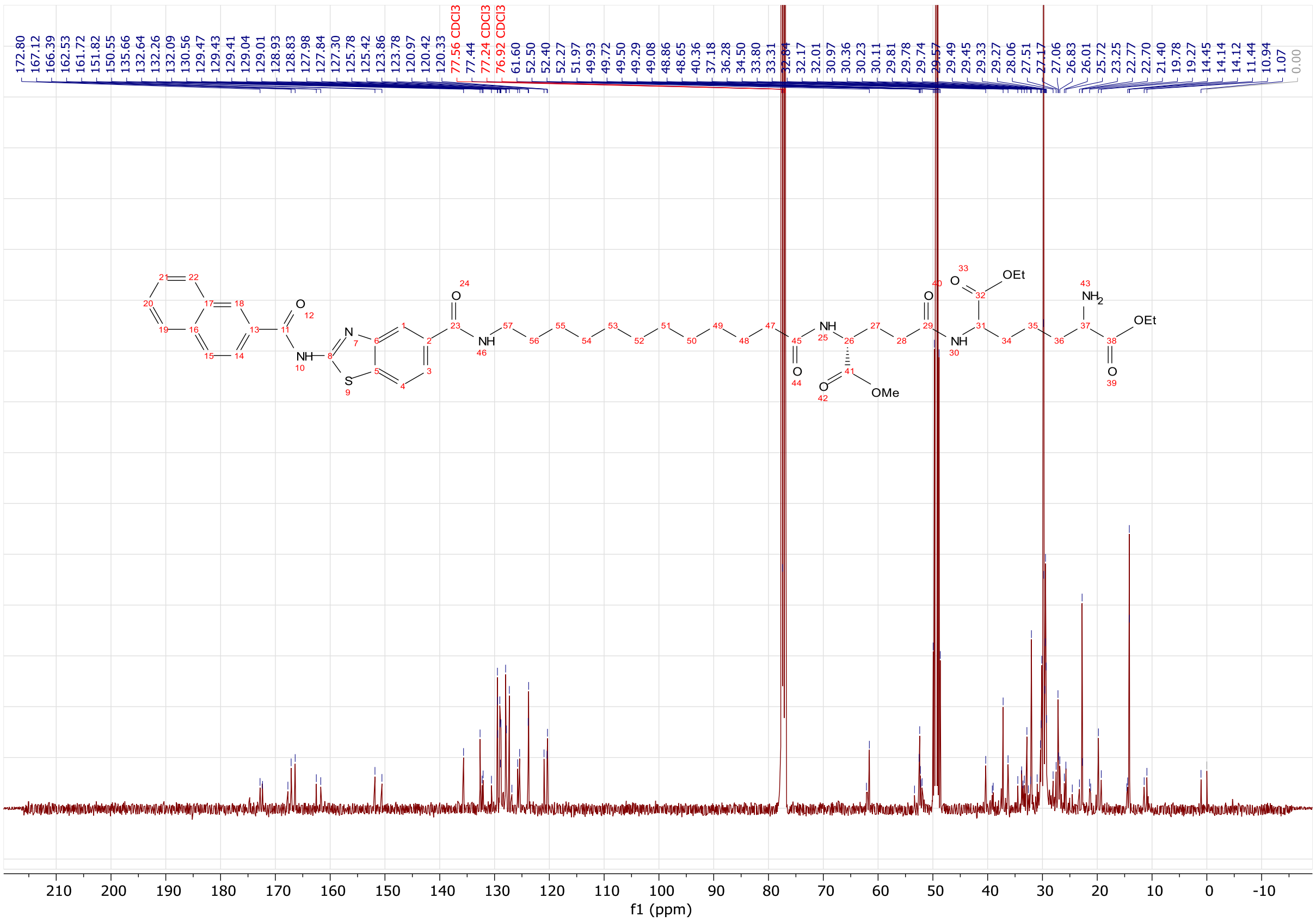

Compound N1/T7: ^{1}H, 400 MHz, $CDCl_3$/MeOD

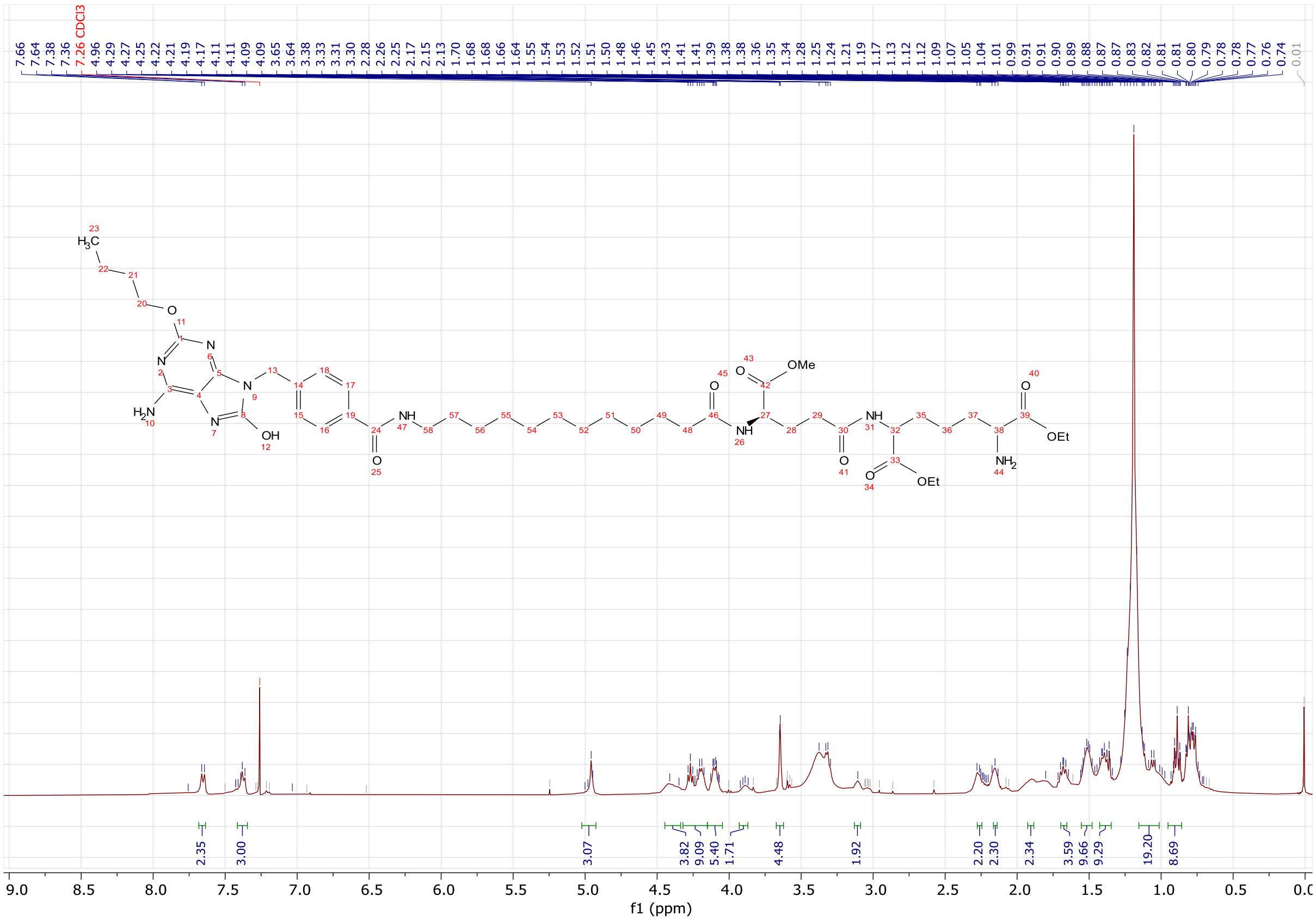

Compound N1/T7: ^{13}C, 400 MHz, $CDCl_3$/MeOD

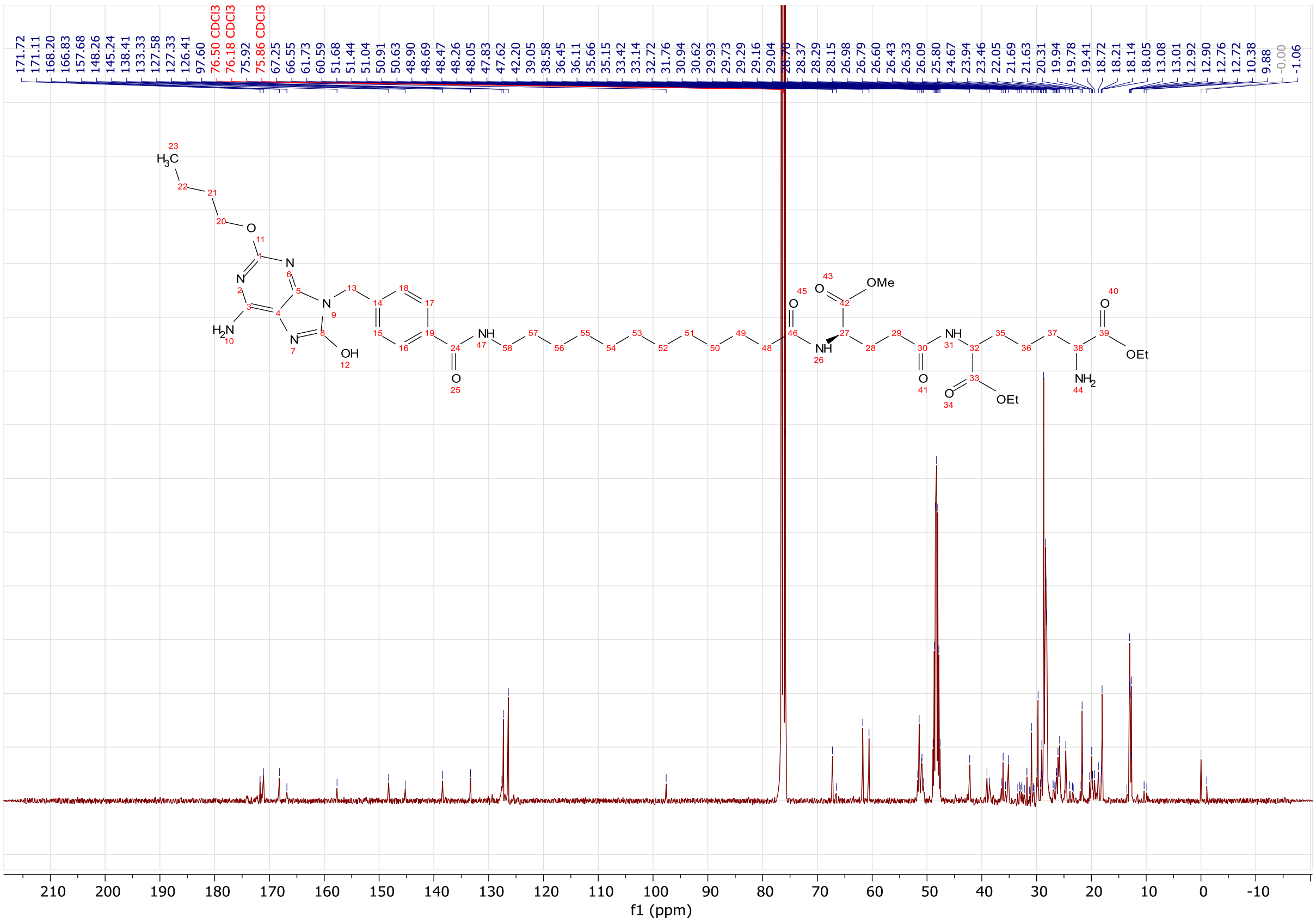

2. UHPLC traces of final compounds

Compound N1/T4:

Chromatogram and Results

Injection Details

Processing Method:	**Quantitative**	*Run Time (min):*	**12,50**
		Wavelength:	**254**

Chromatogram

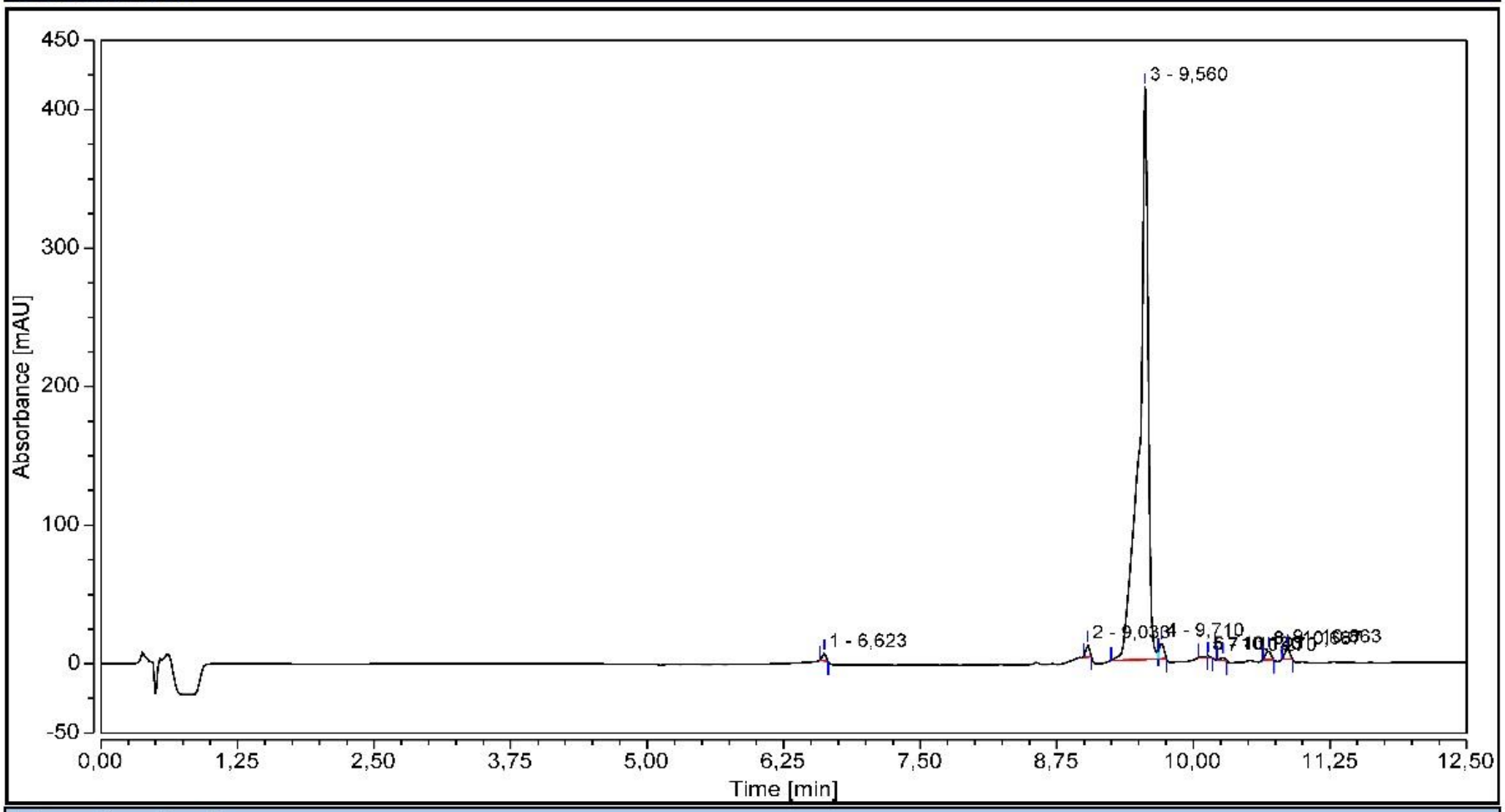

Integration Results

No.	Peak Name	Retention Time min	Area mAU*min	Height mAU	Relative Area %	Relative Height %	Amount n.a.
1		6,623	0,219	5,471	0,54	1,20	n.a.
2		9,033	0,342	8,742	0,84	1,91	n.a.
3		9,560	38,818	413,251	95,04	90,35	n.a.
4		9,710	0,542	11,566	1,33	2,53	n.a.
5		10,130	0,049	1,087	0,12	0,24	n.a.
6		10,143	0,040	1,249	0,10	0,27	n.a.
7		10,270	0,101	2,302	0,25	0,50	n.a.
8		10,687	0,351	6,346	0,86	1,39	n.a.
9		10,863	0,381	7,363	0,93	1,61	n.a.
**Total:**			**40,842**	**457,377**	**100,00**	**100,00**	

Compound N1/RI:

Chromatogram and Results

Injection Details			
Processing Method:	**Quantitative**	*Run Time (min):*	**8,00**
		Wavelength:	**254**

Chromatogram

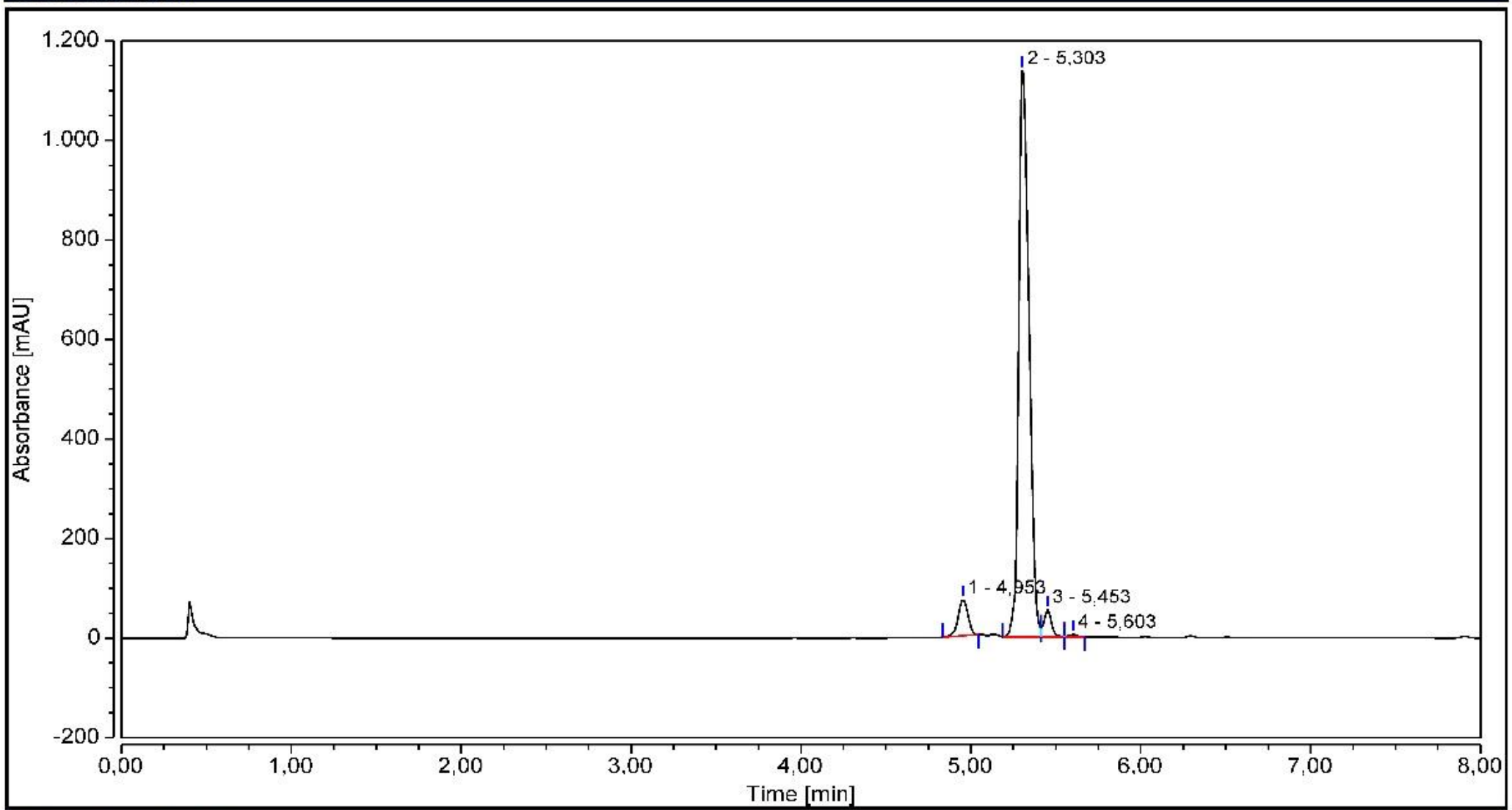

Integration Results

No.	Peak Name	Retention Time min	Area mAU*min	Height mAU	Relative Area %	Relative Height %	Amount n.a.
1		4,953	2,284	72,001	2,60	5,67	n.a.
2		5,303	84,210	1137,862	95,82	89,53	n.a.
3		5,453	1,325	55,469	1,51	4,36	n.a.
4		5,603	0,152	5,616	0,17	0,44	n.a.
**Total:**			**87,884**	**1270,948**	**100,00**	**100,00**	

Compound N1/T7:

Chromatogram and Results

Injection Details			
Processing Method:	**Quantitative**	*Run Time (min):*	**8,00**
		Wavelength:	**254**

Chromatogram

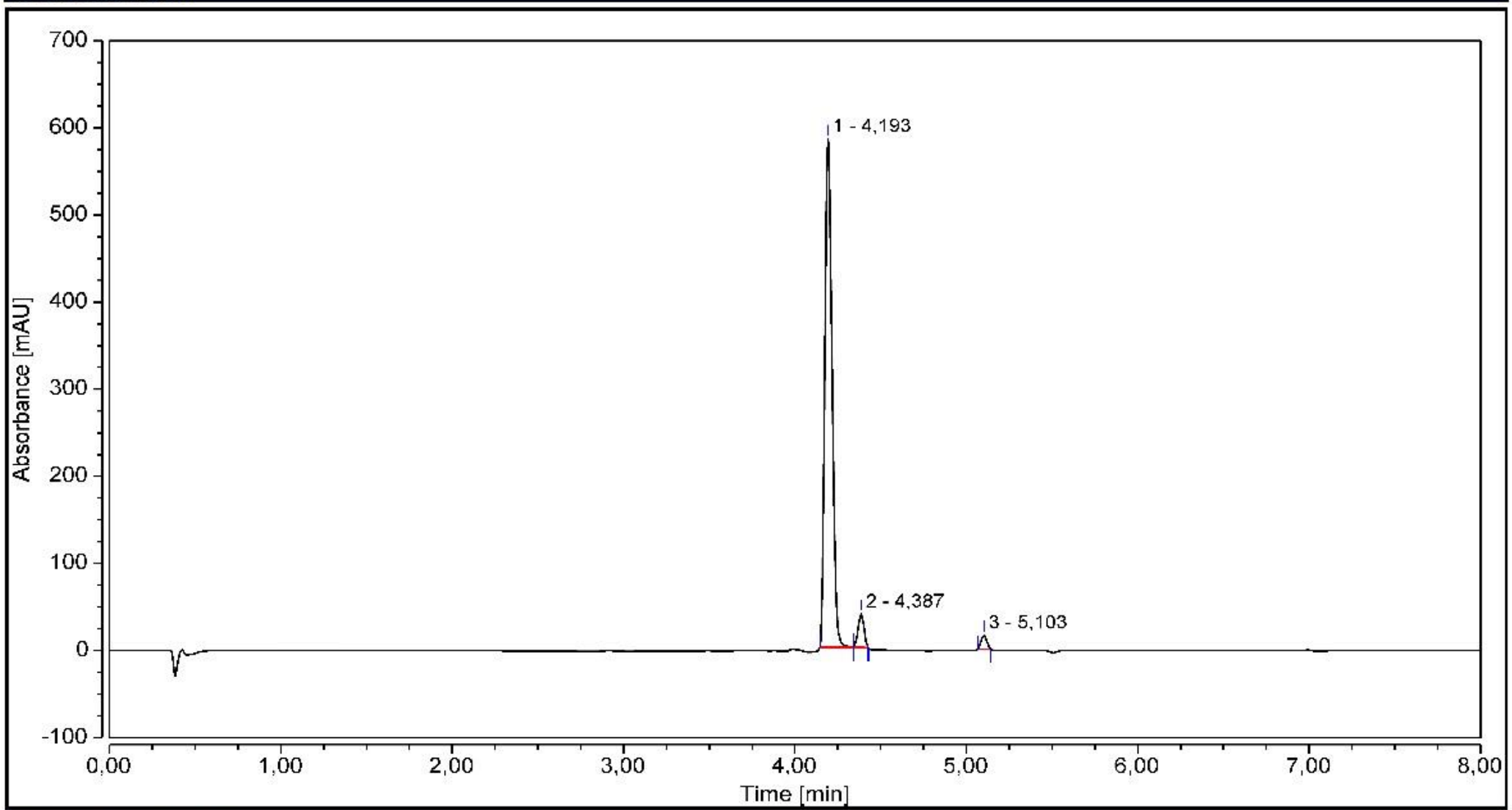

Integration Results

No.	Peak Name	Retention Time min	Area mAU*min	Height mAU	Relative Area %	Relative Height %	Amount n.a.
1		4,193	29,293	583,866	95,90	91,44	n.a.
2		4,387	0,892	38,246	2,92	5,99	n.a.
3		5,103	0,361	16,399	1,18	2,57	n.a.
**Total:**			**30,545**	**638,511**	**100,00**	**100,00**	

3. HRMS of final compounds

Compound N1/T4:

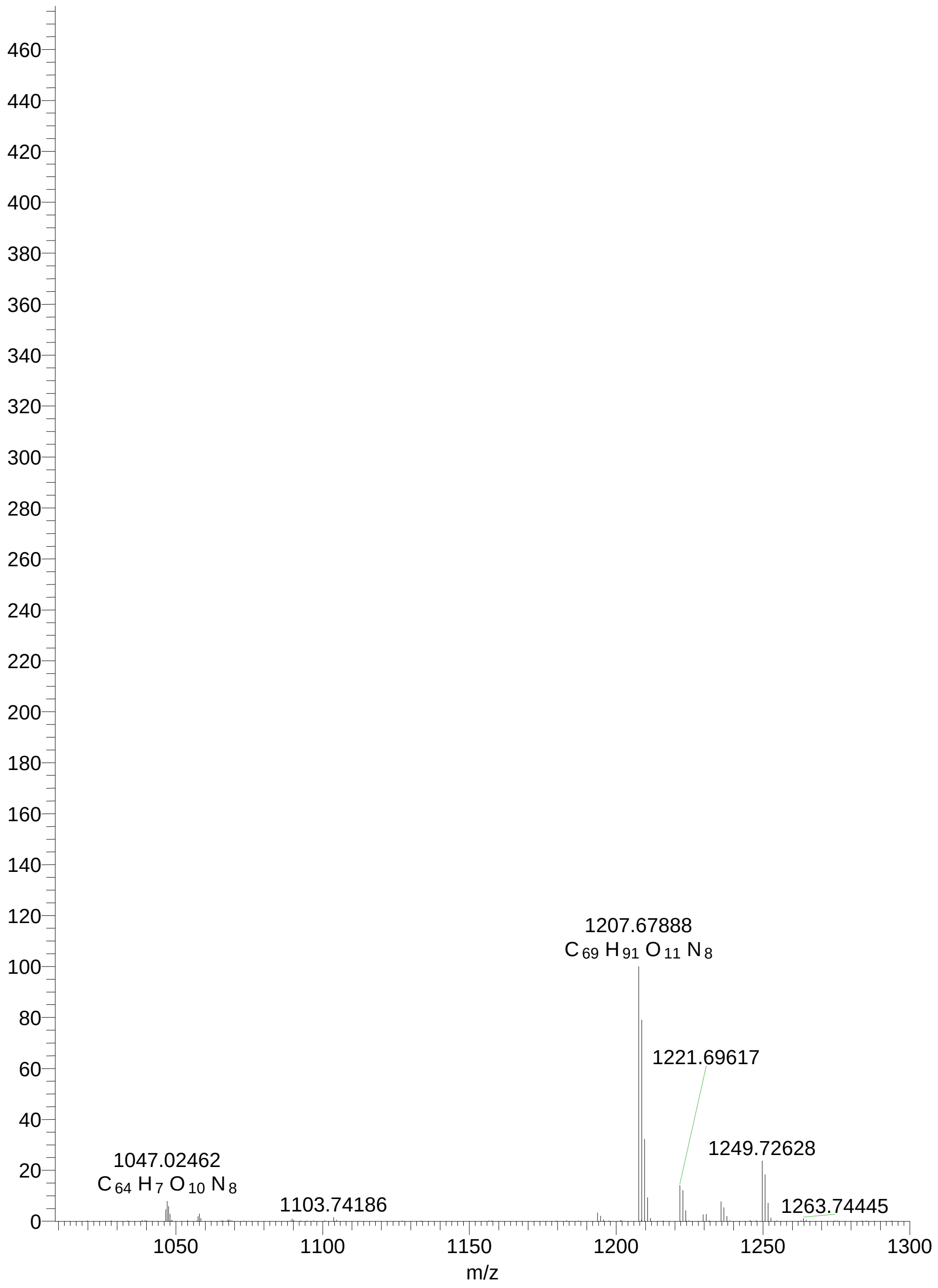

Elemental composition search on mass 1207.67888

m/z= 1202.67888-1212.67888

m/z	Theo. Mass	Delta (ppm)	RDB equiv.	Composition
1207.67888	1207.68018	-1.08	28.5	$C_{69}H_{91}O_{11}N_8$

Compound N1/RI:

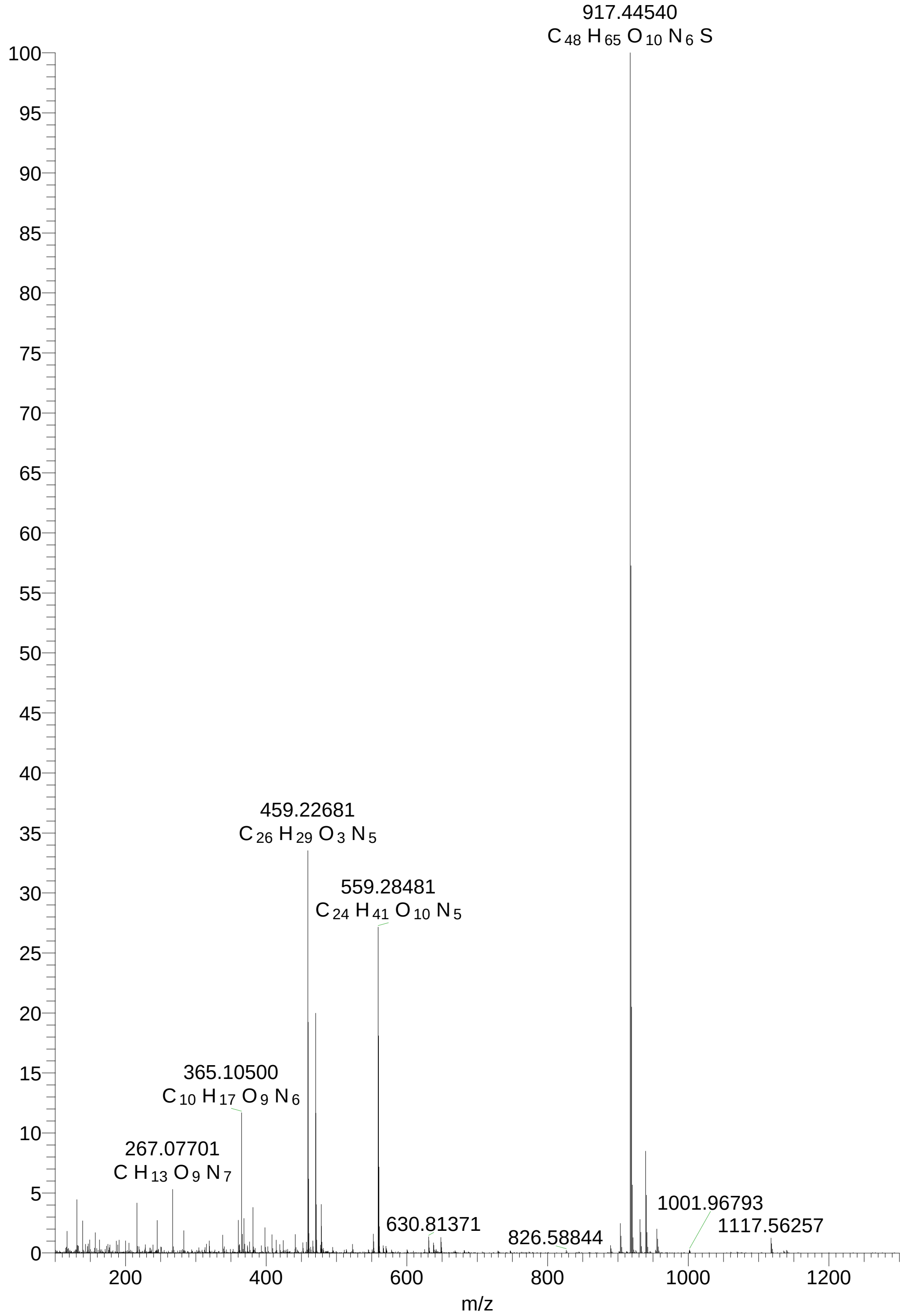

Elemental composition search on mass 917.44540

m/z= 912.44540-922.44540

m/z	Theo. Mass	Delta (ppm)	RDB equiv.	Composition
917.44540	917.44774	-2.55	19.5	$C_{48}H_{65}O_{10}N_6S$

Compound N1/T7:

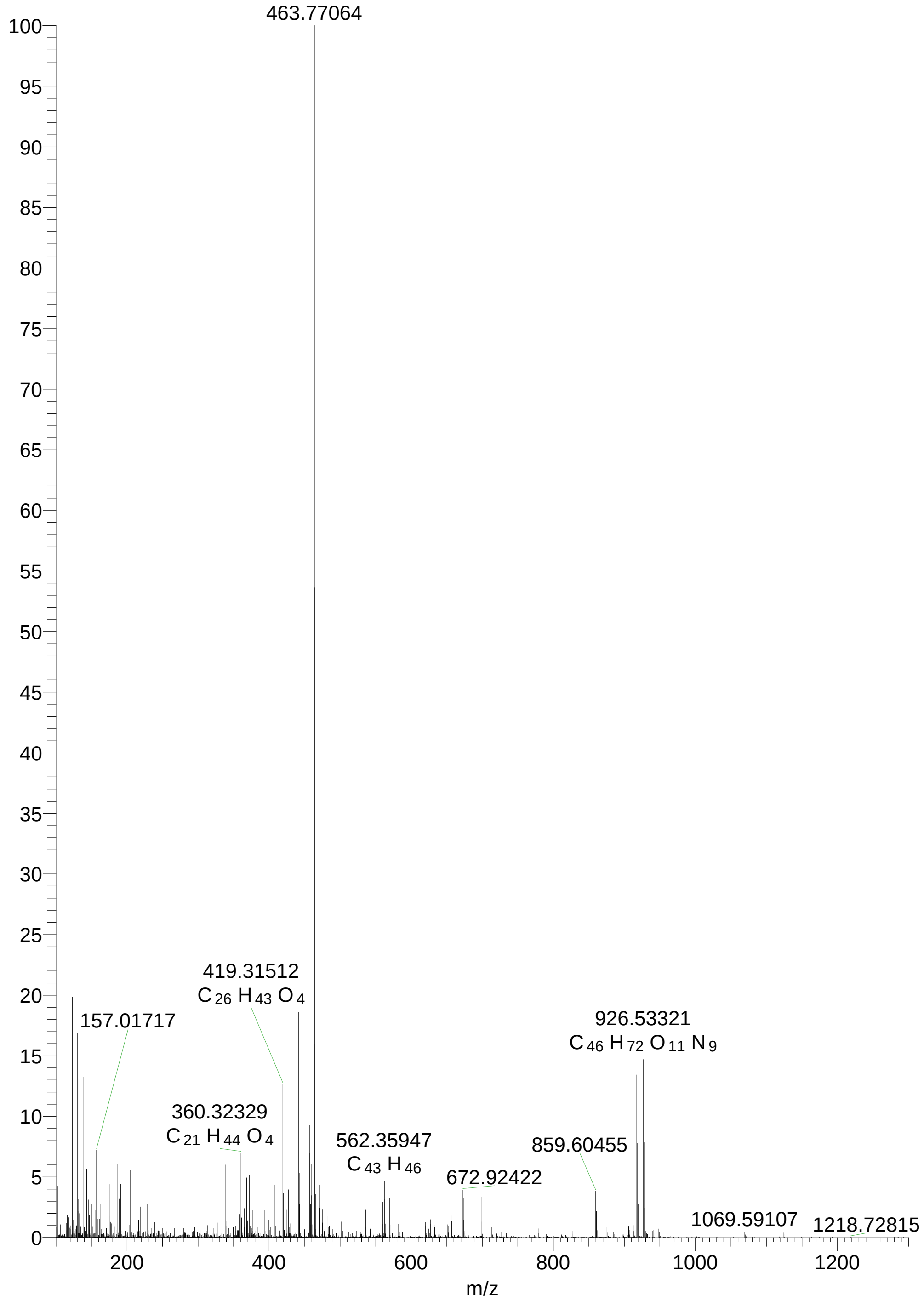

Elemental composition search on mass 926.53321

m/z= 921.53321-931.53321

m/z	Theo. Mass	Delta (ppm)	RDB equiv.	Composition
926.53321	926.53458	-1.48	15.5	$C_{46}H_{72}O_{11}N_{9}$

4. Supporting Figures

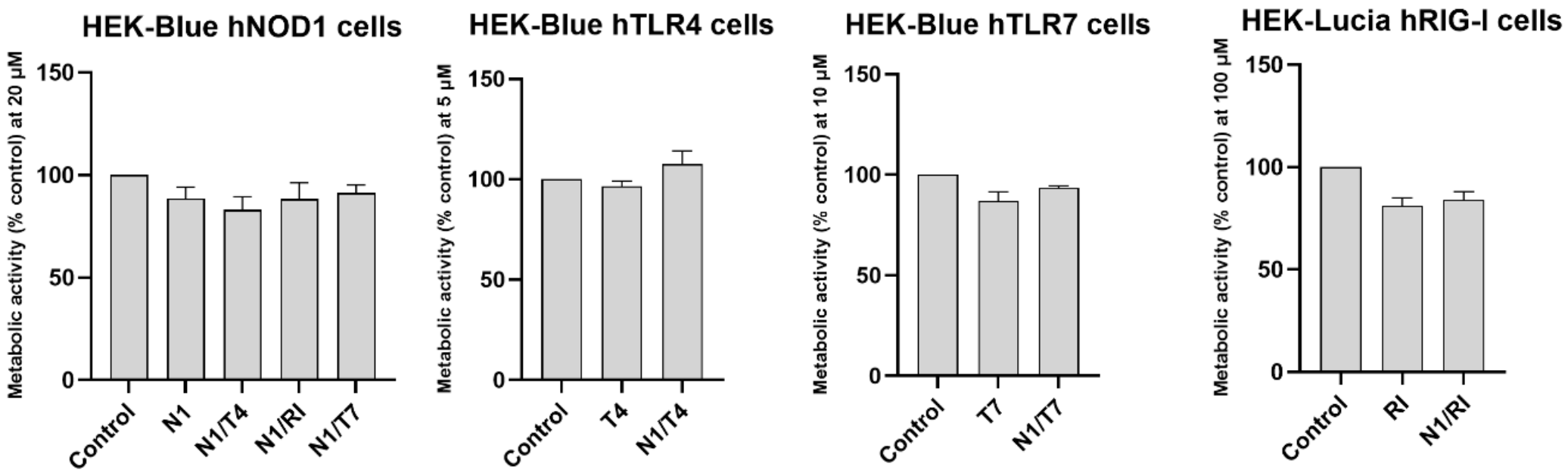

Figure S1. Cytotoxicity profiling: metabolic activities/proliferation rates of HEK-Blue hNOD1, HEK-Blue hTLR4, HEK-Blue hTLR7, and HEK-Lucia RIG-I were measured after 18 h treatment with compounds at indicated concentrations (depending on the highest concentration used for the ensuing assays). Data are shown relative to the untreated control (0.1% DMSO). Data are means ±SEM of two independent experiments (performed in duplicates); ns, not significant versus control (one-way ANOVA post hoc Dunnett's tests).

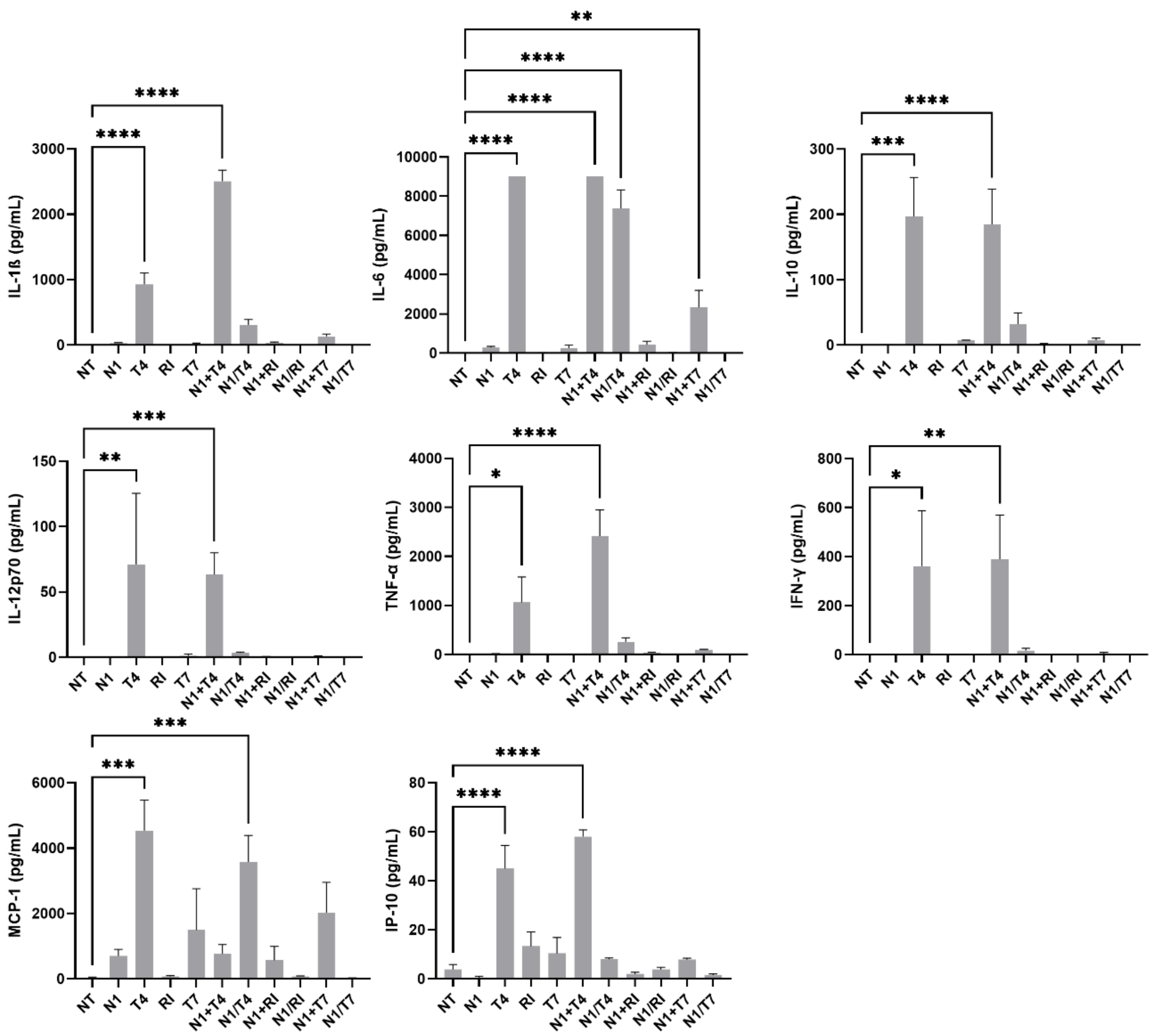

Figure S2. Cytokine profiling of PBMCs stimulated with tested compounds at 1 µM. Cytokine concentrations were measured after 18 h stimulation with unlinked mixtures of corresponding agonists, conjugated agonists or control vehicle (0.1% DMSO). Data are expressed as mean ± SEM of 3 independent experiments. *, $p \leq 0.05$, **, $p \leq 0.01$, ***, $p \leq 0.001$, ****, $p \leq 0.0001$ versus control (one-way ANOVA post hoc Dunnett’s tests).
